# Chaotic and stochastic dynamics of epileptiform-like activities in sclerotic hippocampus resected from patients with pharmacoresistant epilepsy

**DOI:** 10.1371/journal.pcbi.1010027

**Published:** 2022-04-13

**Authors:** Noemi S. Araújo, Selvin Z. Reyes-Garcia, João A. F. Brogin, Douglas D. Bueno, Esper A. Cavalheiro, Carla A. Scorza, Jean Faber

**Affiliations:** 1 Department of Neurology and Neurosurgery, Federal University of São Paulo (UNIFESP), São Paulo, São Paulo, Brazil; 2 Departamento de Ciencias Morfológicas, Facultad de Ciencias Médicas, Universidad Nacional Autónoma de Honduras, Tegucigalpa, Honduras; 3 Department of Mechanical Engineering, São Paulo State University (UNESP), School of Engineering of Ilha Solteira, Ilha Solteira, São Paulo, Brazil; 4 Department of Mathematics, São Paulo State University (UNESP), School of Engineering of Ilha Solteira, Ilha Solteira, São Paulo, Brazil; Newcastle University, UNITED KINGDOM

## Abstract

The types of epileptiform activity occurring in the sclerotic hippocampus with highest incidence are interictal-like events (II) and periodic ictal spiking (PIS). These activities are classified according to their event rates, but it is still unclear if these rate differences are consequences of underlying physiological mechanisms. Identifying new and more specific information related to these two activities may bring insights to a better understanding about the epileptogenic process and new diagnosis. We applied Poincaré map analysis and Recurrence Quantification Analysis (RQA) onto 35 *in vitro* electrophysiological signals recorded from slices of 12 hippocampal tissues surgically resected from patients with pharmacoresistant temporal lobe epilepsy. These analyzes showed that the II activity is related to chaotic dynamics, whereas the PIS activity is related to deterministic periodic dynamics. Additionally, it indicates that their different rates are consequence of different endogenous dynamics. Finally, by using two computational models we were able to simulate the transition between II and PIS activities. The RQA was applied to different periods of these simulations to compare the recurrences between artificial and real signals, showing that different ranges of regularity-chaoticity can be directly associated with the generation of PIS and II activities.

## Author summary

Temporal lobe epilepsy (TLE) is the most prevalent type of epilepsy in adults and hippocampal sclerosis is the major pathophysiological substrate of pharmaco-refractory TLE. Different patterns of epileptiform-like activity have been described in human hippocampal sclerosis, but the standard analysis applied to characterize the activities usually do not consider the nonlinear features that epileptiform patterns exhibit. Here, using Poincaré map and Recurrence Quantitative Analysis we characterized the most prevalent type of epileptiform-like activities—interictal-like events (II) and periodic ictal spiking (PIS), recorded in vitro from resected hippocampi of pharmacoresistant patients with TLE—according to their levels of stochasticity, chaoticity and determinism. The II activities showed to be more chaotic with complex rhythmicity than PIS activities. The nonlinear dynamic differences between II and PIS leads us to conjecture that they are expressions of different seizure susceptibility. We also identified that each hippocampal subfield expresses II and PIS activities in a specific and different way. Finally, from the modulation of internal parameters of two computational models, we show the conversion of one type of activity into the other, showing how specific neuron networks synchronize over time, leading to II and PIS activities and then into a generalized seizure.

## Introduction

Temporal lobe epilepsy (TLE) is the most prevalent type of epilepsy in adults and hippocampal sclerosis is the major pathophysiological substrate of pharmaco-refractory TLE [1–3]. In these cases, neurosurgery for the resection of the affected hippocampus is the best treatment option. Although any surgery is a very complex procedure, it provides an opportunity to investigate the dynamics of epileptiform activity associated with the epileptic brain tissues through in vitro electrophysiology techniques [4–6].

Different patterns of epileptiform-like activity have been described in human hippocampal sclerosis: (i) interictal-like events, (ii) periodic ictal spiking, (iii) seizure-like events and (iv) spreading depression-like events [7, 8]. However, the temporal signal analysis employed to characterize these associated patterns usually do not consider statistical attributes such as high recurrence, non-stationary periods, emerging events from high synchronicity etc. that epileptiform activities exhibit. All these characteristics are typical in nonlinear systems and, thus, difficult to describe using traditional methods [9–12].

Epilepsy represents one of the most critical contexts to investigate nonlinear dynamics associated with electrophysiological patterns since epileptic seizures are natural phenomena produced by hypersynchrony with temporal transitions of equilibrium to non-equilibrium states (and vice-versa) [13]. These characteristics are typically described by chaotic dynamics [14, 15], which are deterministic activities, but can nonetheless manifest under stochastic environment-coupling [16–18]. Indeed, Hayashi et al. [19] showed that induced epileptiform activities in hippocampal slices in the CA3 region of animal models can present local field potentials with chaotic behavior. Babloyantz and Destexhe [20, 21] showed that EEG signals recorded during an epileptic seizure could be characterized as a chaotic system, with small fractal dimension and positive Lyapunov exponent. Other authors reported the presence of chaotic dynamics in an EEG recording during a mixed generalized seizure in patients with TLE [13, 14, 22–27].

Studies of in vitro electrophysiological patterns from hippocampus tissues report that most activities recorded from hippocampal formation tissues from TLE patients present predominantly two types of patterns (> 70%): the interictal-like events (II) and the periodic ictal spiking (PIS) [28]. In this way, although brain slices do not present the full complexity of a whole brain system, the study of epileptiform patterns recorded from hippocampal slices can help to better understand the electrophysiological dynamics generated by sclerotic regions.

The better understanding of these patterns is particularly relevant due to their high incidence and similarity with interictal spiking patterns of intracranial electroencephalography [29, 30]. Until now, these two epileptiform activities have been characterized and distinguished only by their event rates (r), where interictal-like events (II) present *r* < 40/*min*, and periodic ictal spiking (PIS) presents *r* > 40/*min* [7, 8]. Therefore, two important points are whether these event rates carry information about the epileptogenic process, and whether they hide nonlinear features related to epileptogenic mechanisms [19, 31–36]. Most importantly, there are no studies reporting whether the differences between II and PIS are sustained on each hippocampal (HP) subfield or whether it is a general HP activity. If so, what is the relationship between the dynamics presented by these patterns and the specific hippocampus circuitry of each HP subfield? In this sense, our first hypothesis is there might be different ‘hidden dynamics’ related to each one of epileptiform patterns, II and PIS, which cannot be assessed based only on visual inspection and spiking rate classification. Our second hypothesis is that these statistical ‘hidden dynamics’ are promoted by intrinsic physical properties associated to the epileptogenic process.

To address these issues, we applied the Poincaré map and the Recurrence Quantitative Analysis (RQA) techniques that are two systematic procedures that map geometric features of a time series onto its phase-space [37, 38]. While the Poincaré Map provides information on the variability of the inter-event intervals, the RQA provides a set of factors that capture the essence of stochasticity, determinism and chaoticity dynamics associated with the epileptiform patterns [39].

By using the Poincaré map and RQA, we were able to quantify nonlinear factors associated with II and PIS, recorded in vitro from resected hippocampi from pharmacoresistant patients with TLE. Poincaré maps revealed that the interspike intervals of II and PIS contain relevant information and it can be distinguished, implying that the way epileptic spikes are discharged in time must be considered. By analyzing both skewness and kurtosis, we also found that each hippocampal subfield expresses II and PIS patterns differently.

As for the RQA coefficients, we found that the temporal event recurrences were the main factor for distinguishing II from PIS. II activities most probably come from a coupled system, exhibiting a predominantly chaotic and complex rhythmicity (with stochastic fluctuations), whereas PIS patterns proved to be predominantly non-chaotic deterministic (with stochastic fluctuations). The nonlinear dynamic differences found between II and PIS lead us to conjecture that II and PIS are expressions of mediums with different seizure susceptibilities, possibly due to different epileptogenic stages. We also identified that each hippocampal subfield exhibits specific II and PIS patterns, which can be interpreted as a consequence of the specific neuronal circuitry associated with each subfield. Stochastic and chaotic dynamics were mostly found in the dentate gyrus (DG) and subiculum, while epileptiform-like activities with low levels of chaoticity and stochasticity were mainly encountered in CA1-CA4.

These results together suggest that the differences between II and PIS patterns are related to a variation of the epileptic sclerosis effect, such as abherrant networks, biochemical imbalance and/or structural network rupture. To support these conjectures, we also implemented two well-known computational models: the Hindmarsh-Rose (HR) model [40], and the Izhikevich (IZ) model [41]. By changing specific parameters, it was possible to modulate their simulated activities from a more regular to a more chaotic pattern, approximating their statistical features to the PIS and II patterns. The RQA was applied to different periods of these simulations to compare the recurrences measures between artificial and real signals to capture how the transition from one pattern to the other may occur.

The results show that different ranges of regularity-chaoticity, separated by a smooth function that captures their transition, can be defined and linked to the generation of PIS and II activities. Furthermore, considering a complementary analysis involving autoregressive models and logistic regression applied to the time series and computational models, a good adherence between real and artificial activities was verified, thus endorsing the representativeness of such models to II and PIS patterns.

## Materials and methods

### Ethics statement

The study was approved by the Ethics Committee of Universidade Federal de São Paulo (CAAE 47551015.1.0000.5505 and / data processing: CAAE 7961030418) and written informed consent was obtained from all patients.

### Electrophysiological *in vitro* recordings

We analyzed epochs with 148 seconds of 35 electrophysiological data recorded from slices of 12 human hippocampal specimens surgically resected from patients with pharmacoresistant TLE (details of clinical features in Fig A in S1 Text).

After hippocampal resection, the tissue was coronally dissected into slices with thickness of 500 *μm* using a vibratome (Leica VT 1200S). The slices were transferred to an interface chamber with a perfusion rate of 1.7–2.0 mL/min with prewarmed (34.5 ± 0.5 °*C*) carbonated artificial cerebrospinal fluid (aCSF). Extracellular recordings started after a recovery period of 4–5 hours from the slice preparation. See Reyes-Garcia and colleagues [28] for details of the slice recodings and ictal patterns categorization [42].

Recordings were performed in the granule cell layer of the dentate gyrus and in the pyramidal cell layer of the CA1, CA2, CA3, CA4 and subiculum, by using glass electrodes filled with 154 mM NaCl, placed 150 *μm* below the surface of the slice. The epileptiform activity was induced in the subiculum and CA1-CA4 subfields with continuous aCSF perfusion containing 10–12 mM [K+] (high K-aCSF). In the dentate gyrus, hilar stimulation and continuous perfusion with high K-aCSF were required to induce epileptiform activity [7, 8, 28].

### Electrophysiological signals processing

**Fig 1:**
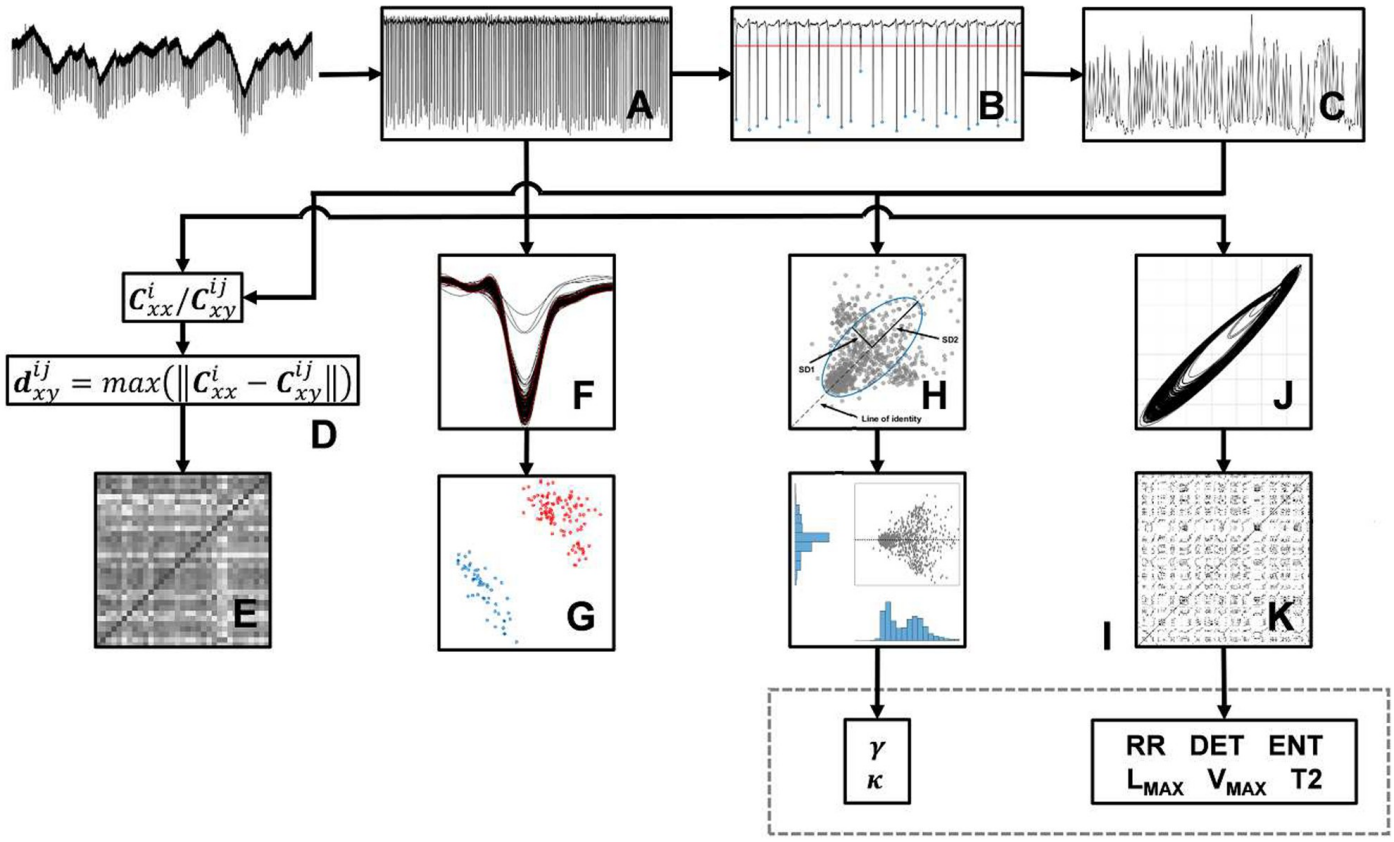
General scheme of the methodology. (A) The analysis started with raw data filtering for 60 Hz and harmonics removal. (B) Next, we did an automatic event-spikes detection, considering a threshold of 0.1mV and (C) calculated the interspike intervals *T*_*n*_ (in seconds). (D) For the correlation analysis, we calculated the autocorrelation and cross-correlation from the time series in (A) and (C). (E) Next, we calculated the distance $d_{xy}^{ij}$ between autocorrelation **C**_*xx*_ and cross-correlation **C**_*xy*_ by Kolmogorov-Smirnov test statistic, obtaining two square matrices, one from time series in (A) and another from time series in (C). (F) To sort the waveforms, we applied a centralization into all event-spikes of the electrophysiological recordings in (A), using their minimum as reference. (G) Then, we used PCA to identify clusters and comparing the correlations between the waveform morphologies of II and PIS activities. (H) To first way of nonlinear analysis, we reconstructed the Poincaré map plotting successive intervals *T*_*n*_ versus *T*_(*n*+1)_ and we fitted an ellipse to the points of the map, with its center determined by the average of the intervals. (I) Next, we evaluated the shape of the histograms distribution from the Poincaré map points projected onto the line of identity and onto its orthogonal axis and calculated the skewness and kurtosis of these histograms. (J) Finally, before the second way of nonlinear analysis by RQA, the electrophysiological recordings in (A) were down-sampled at 1 kHz, to construct phase space for each record, (K) then the Recurrence Plot and subsequent quantification analysis was done. All phase space was reconstructed with embedding dimension *m* = 3 and time delay *τ* = 1. To RQA, was applied *l*_*min*_ = 3 and *v*_*min*_ = 3 to minimum length of diagonal and vertical lines, respectively, and a Theiler window of width 2 (*t*_*w*_ = 2).

The electrophysiological signals were recorded at 10 kHz and amplified with a gain of 20x using a custom-made amplifier, digitized on-line and stored for off-line analysis using Spike 2 v6.09 (CED-1401, United Kingdom). Except where otherwise indicated, all analysis was carried out by customized scripts running in MATLAB (version 9.2.0 R2017a, Mathworks Inc., MA, USA). All data sets were processed using an infinite impulse response (IIR) notching comb filter with order 167 and Q factor 35 to remove 60 Hz and harmonics (Fig 1A).

### Series of interspike intervals

We developed an algorithm for automated detection of interictal-like events and periodic ictal spiking along the time series recordings for each hippocampal subfield (subiculum, CA1, CA2, CA3, CA4, and dentate gyrus). Through this algorithm we were able to identify the epileptiform-like events based on a threshold crossing detector, considering a threshold of 0.1 mV (Fig 1B). All automated event detections were visually evaluated by an expert and the false detections were excluded. The electrophysiological signals were classified between interictal-like events or periodic ictal spiking according to their event rates [7, 8]. After the events identification and categorization, using spikes as time markers [34], we constructed the series of event time intervals *T*_*n*_, also called interspike intervals (Fig 1C).

### Correlation analysis

To evaluate correlations among the recorded types of epileptiform activity, we calculated the autocorrelation $C_{xx}^{i}=\text{xcorr}\left(x_{i},x_{i}\right)$ and the cross-correlation $C_{xx}^{ij}=\text{xcorr}\left(x_{i},x_{j}\right)$, considering all series of interspike intervals and all extracellular recordings (*x*, *y*) for each hippocampal subfield (*i*, *j*) [19]. Afterwards, we determined which cross-correlations were closer to their respective autocorrelation by calculating their maximum distance through the metric used by the Kolmogorov-Smirnov Test [43] − this procedure yields a square matrix where each element (*i*, *j*) corresponds to an asymmetric index of dissimilarity given by $d_{xy}^{ij}=\text{max}\left(∥C_{xx}^{i}-C_{xy}^{ij}∥\right)$, with $d_{xy}^{ij}\neq d_{yx}^{ij}$, ∀ *i*, *j* (Fig 1D and 1E). It means that high values of $d_{xy}^{ij}$ correspond to distant values of $C_{xy}^{ij}$ from $C_{xx}^{i}$, providing a relative index of dissimilarity. Finally, we averaged all the distance correlation trials associated with the same i-hippocampal subfield, but distinguishing the two types of epileptiform activities, II and PIS, in order to see whether any relevant effect between them would appear.

We also investigated the correlations between the waveform morphologies of II and PIS activities, through a spike-sort-like procedure [44]. To do so, we considered a signal window of 100 ms, cut from each electrophysiological recording, referenced and time-locked using the minimum peak as a reference of the epileptiform event (Fig 1F). Next, once all cut-signal superposed, Principal Component Analysis (PCA) was applied on these windows (Fig 1G) to identify clustered differences and similarities among the types of epileptiform-like activities, II and PIS [45].

### Poincaré map

To construct the Poincaré map, we considered the interspike interval series and the time *T*_*n*_ versus *T*_(*n*+1)_ plot (Fig 1H). The scatter of state points (*T*_*n*_, *T*_*n*+1_) displayed represents an orthogonal cut on the original trajectory flows on the phase-space [34] and is a conventional way to visually represent nonlinear dynamics [19].

To quantitatively characterize the dispersion patterns associated with the Poincaré map of each epileptiform activity, II and PIS, we calculated dispersion factors by applying the ellipse-fitting technique. In this approach, an ellipse is fitted according to the dispersion of the points onto the scatter map, where its centroid is determined by the average of the interspike intervals. The minor axis of the ellipse quantifies the standard deviation of the interspike intervals (SD1) and the major axis quantifies the standard deviation of the successive difference of interspike intervals (SD2) [46]. Thus, SD1 reflects the instantaneous variability of the interspike intervals, which quantifies the abrupt variation in short-term time scale, and SD2 reflects the continuous variability of the interspike intervals, representing the overall variation [47, 48].

Additionally, we also calculated the skewness *γ* and kurtosis *κ* [49–51] associated with the histograms calculated from the projections of the Poincaré map points onto the line of identity (45° line) and onto its orthogonal axis [46, 48], as shown in Fig 1I.

### Recurrence quantification analysis (RQA)

RQA quantifies nonlinear features through factors that evaluate the dynamics of a signal in terms of periodicity, regularity, coupling and noise, over time [52, 53]. To apply this approach, we down-sampled all electrophysiological recordings to 1000 Hz and filtered the fast oscillations at 50 Hz with a least-square order 50 low-pass filter. To estimate the minimum embedding dimension (*m*) of the data, we applied the method of False Nearest Neighbors (FNN) [34, 53]. The time delay (*τ*) was defined according to the mutual information of the time series [54]. Additionally, we used all data sets to define the minimum embedding dimension and the time delay. Then, to plot the trajectories of the attractors, we constructed the phase space (Fig 1J) by using the method of delays [53, 55]. To have a robust statistical effect on the analysis of RQA factors, we selected 15 periods of 10 seconds along each electrophysiological recording.

RQA is based on the evaluation of dot-structures presented on the Recurrence Plot (RP) **R**_*i*,*j*_ (Fig 1K) through different nonlinear factors [37, 53]. Each dot (*i*, *j*) of the RP is calculated according to the recurrence rule, described by **R**_*i*,*j*_ = 1, if $∥{\vec{x}}_{i}-{\vec{x}}_{j}\leq \epsilon ∥$, and **R**_*i*,*j*_ = 0 otherwise; where ∥.∥ is the Euclidian distance metric, *ϵ* is the recurrence threshold and ${\vec{x}}_{k}$ are vectors pointing to the amplitude coordinates in the phase space (*t*_*k*_,*t*_*k*+*τ*_,…,*t*_*k*+(*m*−1)*τ*_). Since the columns and rows of a RP correspond to temporal coordinates of the time series, the elements of the matrix express each moment that a relevant state (according to a proper recurrence threshold *ϵ*) of a dynamic system repeats along time [37, 52, 56]. Since the electrophysiological signals exhibit complex and unknown patterns along time, we performed an exhaustive analysis on the recurrence threshold, to evaluate whether there would be optimal regimes associated with both epileptiform-like activities, II and PIS, and with each hippocampal subfield. This analysis was made by means of calculating each RQA factor with different values of *ϵ* [37]. To minimize the effects of tangential motion on the phase space trajectories, associated with the smoothing of the data, we applied the Theiler correction [57] and excluded the line of identity and their neighbor diagonals from all RPs.

The recurrence factors quantify dot-line patterns on RP matrices **R**_*i*,*j*_ [37], given a specific threshold *ϵ*. Since diagonal lines represent the number of signal-event recurrences, the longer the diagonal lines are in **R**_*i*,*j*_, the higher the deterministic degree of the system is. Hence, a pure stochastic signal, such as white noise, does not exhibit diagonal lines, only spuriously. Chaotic systems, on the other hand, are coupled-deterministic systems [10, 58], sensitive to initial conditions, showing a signal behavior expressed by atypical configurations of big diagonal lines in **R**_*i*,*j*_ [37]. Here, we considered the following recurrence factors [53, 59]: (i) Recurrence rate (RR): quantifies the percentage of recurrence points in the RP. RR essentially measures the capacity of a system to return to the neighborhood (defined by *ϵ*) of a previous state; (ii) Determinism (*DET*): measures the fraction of recurrence points that form diagonal lines parallel to the line of identity of the RP, with a minimum length *l*_*min*_. *DET* is interpreted as the measure of the predictability degree of a system; (iii) Entropy (*ENT*): measures the Shannon entropy of the probability distribution of the diagonal line lengths. *ENT* reflects the complexity of the system, therefore high *ENT* values imply in high diversity of the diagonal lengths (majored by the uniform distribution), reflecting irregular recurrences or complex dynamics; (iv) Maximal diagonal line length (*L*_*max*_): is related to the divergence of the trajectory segments on phase-space. Since diagonal lines are related to the distance between two segments in the attractor trajectories at a different time, the smaller the *L*_*max*_, the more divergent the trajectories are; (v) Maximal vertical line length (*V*_*max*_): vertical lines result from the proximity of a single state to a series of other states in its own trajectory line, reflecting the time scale of small changes along the entire recording. Therefore, *V*_*max*_ is related to the time at which the system stays stuck in a holding pattern; and (vi) Recurrence times of the second type (*T*2): means how long the system remains inside a given event. *T*2 is related to the periodicity of the signal and can be useful to detect changes of the dynamics. Although a high value of *DET* is a necessary condition to detect determinism, it is not sufficient to indicate pure periodicity. However, to increase the reliability of this factor, we stablish a minimal length for a diagonal recurrence (*l*_*min*_) equal to three [53, 60].

The following terminology, associated with RQA factors, will be adopted in this work:

- Deterministic activity: ideal temporal patterns completely determined by one or more independent variables and/or parameters [61]. The mathematical description of deteterministic activities does not exhibit random variables (or factors), and its dynamic is not sensitive to initial conditions [62]. These patterns are associated with well-defined periodic patterns in some temporal scale. The main related RQA fators are high *DET*, high *RR*, and low *ENT* [37];
- Stochastic activity: temporal patterns mathematically determined by time-dependent random variables, random factors and/or noise [63]. These activities often present erratic-event recurrences, governed by some probability distribution [62]. It works in the opposite direction of deterministic activities. The main related RQA fators are low *DET*, low *RR* and high *ENT* [64];
- Chaotic activity: dynamics related to particular type of physical coupling that yields temporal patterns sensitive to initial conditions [62, 65], and erratic patterns not related to any random variable. Strictly, a chaotic pattern is therefore a deterministic activity, although it can also be conjugated with stochastic effects [62]. Depending on the system’s conditions/parameters, it can present emergent phenomena, instability and erratic temporal recurrences bounded to attractor points in its phase-space [62, 66]. The main related RQA fators are high *ENT* and high *L*_max_ (which is associated to the Lyapunov exponent, a classic indicator of chaoticity [67, 68]);
- Regular activity: here we call “regular” temporal patterns strictly associated with deterministic but not chaotic dynamics. Since completely regular (or deterministic) patterns do not exist in real biophysical systems, all quantifications must be relative and gradual. In these terms, “irregular activities” work in the opposite direction of the regular ones, representing any type of erratic pattern, chaotic and/or stochastic;
- Irregular activity: temporal patterns associated with chaotic with (necessarily) some level of stochasticity. Particularly these patterns will be related to non-linear prediction, erratic recurrences, emergent patterns, bifurcations in phase-space [69].

The RQA factors T2 and *V*_max_ [37] must be considered accordingly to each context, observing their mean and variance over time. Depeding on the magnitude of the other factors they can corroborate or not a specific signal behavior. Since none of these labels are absolute themselves, we will often resort to gradations to refer to the behavior of the recorded patterns.

Here, the terms chaoticity, stochasticity and determinism have to be thought in terms of the epileptic acitivities. An epileptic activity is often regarded as a hypersynchronization of neuron discharges over time [13]. However, although synchrony is important and required for a health functioning of brain activity, a hypersynchrony is not (mainly if it is spread over different nucleous and/or brain regions). When a neuronal network is totally synchronized, it lacks complexity because it loses other types of activities, associated to stochastic and chaotic patterns of spiking. In this case, the system presents a more regular activity. On the other hand, if a neuronal network discharges are totally random, without any synchronicity, it also lacks complexity, pointing the system to a more stochastic dynamics. Complexity patterns occur, therefore, in regimes between regular and irregular activities [70]. Nevertheless, since we are evaluating here only epileptiform spikes, the terms regular and complex need to be contextualized, since they refer to the “temporal ordering” of epileptic events − which are related to failure of inhibitory mechanisms [33]. Moreover, we have to consider that stochastic fluctuations are always present in real systems, which is why RQA factors must be analyzed comparatively and statistically, mainly because we do not have control groups.

### Statistical analysis

A one-sample Kolmogorov-Smirnov normality test was applied to determine a correspondent parametric or nonparametric test. For pairwise comparisons of non-parametric tests, a Wilcoxon rank-sum test was used, and for group comparison, a non-parametric analysis of variance (Kruskal-Wallis test) was applied, since the samples did not pass the normality test. The significance level for all analyses was established using *α* = 0.05.

### Numerical simulations of computational models

Numerical simulations were carried out to reproduce the main patterns II and PIS (see S1 Text for a complete description). Two specific neuron dynamic models were selected to represent these activities, namely: the Hindmarsh-Rose model [40] and the Izhikevich model [41]. A more detailed description and explanation about the adequability of these models can be found in S1 Text.

The main idea using these models is to vary specific parameters of each model within a range that assures a transition of activity between chaotic and regular spiking [71, 72]. That is, by showing that two general signals with physical and statistical properties, such as those exhibited by signals II and PIS, based on RQA factors, can be modulated by specific parameters (as those described in the HR and IZ models), it would help to highlight a common origin modified by some (patho)physiological difference. Using these computational models, we show that it is possible to represent a transition from one deterministic periodic type activity to a deterministic chaotic pattern and vice versa, which in this work is modeled by as sigmoid function.

From a biological point of view, although the substracte is the same (statistically speaking), the premise for the transition model is that the epileptic process would produce structural differences capable of modifying the functional patterns. From a physical point of view, in principle, two different patterns can be generated, for example, by modulating coupling or noise parameters, yielding phase transitions [62]. In this way, if we can model two distinct dynamics, preserving the statistical similarities of II and PIS, by modulating a specific parameter of a same model, we can assume that an intrinsic factor (and not a spurious one) is generating these two activities. However, although these two activities come from the same biological structure, given their statistical features, and the lack of concomitant recordings [28], we interpret them as two distinct physical systems, discriminated by the modulation of a parameter that emulates some aspect of the biological structure. The parameters of the computational model intend to minimally represent biophysical quantities related to this modulation [73]. How much a parameter needs to change represents the addressed phase transition given by the sigmoid function.

Finally, the RQA factors were calculated to several epochs of the simulated signals, whose control parameters were set so as to represent different interictal and ictal regions, namely: *II*_1_, *II*_2_, *II*_3_, *II*_4_, *II*_5_, *TR*, *PIS*_1_, *PIS*_2_, *PIS*_3_, *PIS*_4_, *PIS*_5_, where *TR* is a transition. Further details about this step can be found in S1 Text. All simulations were carried out using the fourth-order Runge-Kutta algorithm with a time step of 0.01s, with Python 3.7 on the Spyder plataform, which is free and open-source.

## Results

### Relationship between epileptiform-like activities and a possible common physiological mechanism

**Fig 2:**
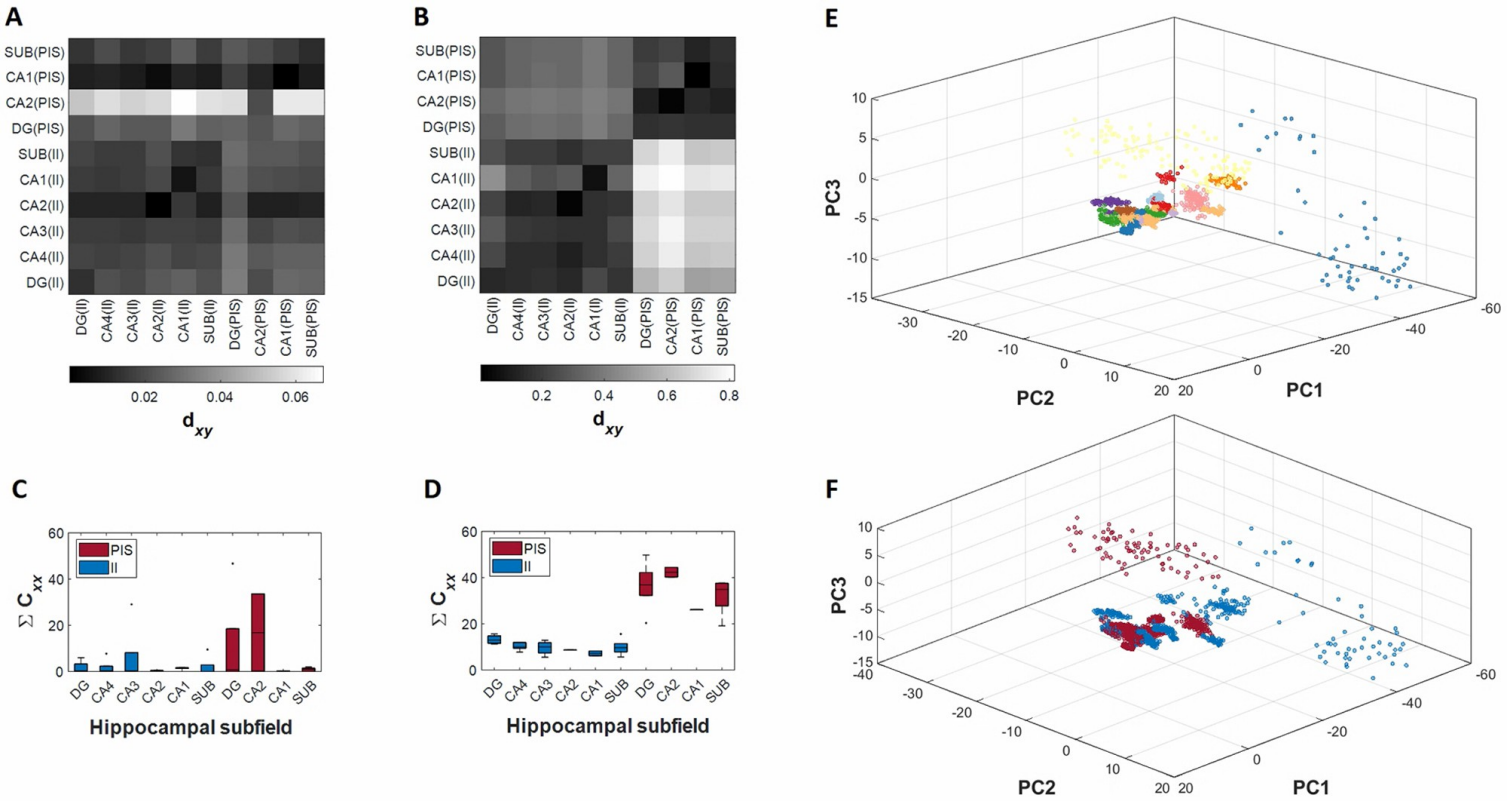
Correlation with a common physiological mechanism. Average distance between autocorrelation **C**_*xx*_ and cross-correlation **C**_*xy*_ from (A) electrophysiological records and from (B) series of interspike intervals. The color bar shows the degree of similarity, with blue indicates more correlation (less distance between **C**_*xx*_ and **C**_*xy*_). Distributions of sum of normalized autocorrelation coefficients from (C) electrophysiological records and from (D) series of interspike intervals. (E) Correlation of waveform morphologies from II and PIS activities with PCA. Each color represents a patient in which the electrophysiological signals was recorded. (F) Same picture as (E) but emphasizing the discrimination of II (blue) and PIS (red) activities.

Fig 2 shows the values of the dissimilarity indices $d_{xy}^{ij}$ quantifying the differences between cross-correlation and autocorrelation, considering all electrophysiological recordings (Fig 2A), and all series of interspike intervals (Fig 2B), respectively. In these graphics, high values of $d_{xy}^{ij}$, displayed with “lighter colors”, correspond to high dissimilarities and low values of $d_{xy}^{ij}$, displayed with “darker colors”, correspond to a low index of dissimilarity among activities. These results show that electrophysiological recordings exhibit less evident information of dissimilarities than interspike interval series, comparing II and PIS activities. Since cross-correlation is a linear metric, the electrophysiological recordings cannot be distinguished linearly, but by mapping them onto the interspike interval space it is possible to linearly distinguish them.

Particularly, the hippocampal subfields presented stable patterns of dissimilarities, regarding II and PIS. However, as shown in Fig 2A and 2B, the PIS activity from CA2 subfield presents divergent high values of $d_{xy}^{ij}$ in comparison to the other hippocampal subfields, especially when considered the electrophysiological recordings directly (Fig 2A). But this effect is attenuated for dissimilarities using interspike intervals.

In addition, by comparing the magnitude of the autocorrelation coefficients, $C_{xx}^{PIS}$ and $C_{xx}^{II}$, as shown in Fig 2C and 2D, we found the first indicative that PIS activity is more periodic than II activity, since their indices $C_{xx}^{PIS}$ are higher than II indices, suggesting the presence of repetitive patterns, linearly displayed. The differences between $C_{xx}^{PIS}$ and $C_{xx}^{II}$ are also more evident and expressive for the interspike intervals than for the recordings. Since autocorrelation represents a self-comparison, along a time-lag, the results indicate again that the possible similarities between II and PIS activities are not linearly detected. However, the interspike intervals easily distinguish II from PIS, not only by rate but also by their event-temporal variability.

More particularly, we also evaluate the similarities among each event-waveform of each epileptiform-like activity from each hippocampal subfield by applying a spike-sort like procedure (Fig 2E). This analysis focuses on linear similarities in a fine temporal scale, assuming that each event itself carries some distinct information according to their activity or subfield. However, as shown in Fig 2E, the waveforms were not grouped according to types of epileptiform activity, neither according to subfield features, but mainly according to the patient’s characteristics. Nevertheless, since this approach evaluates covariance, it also indicates that possible differences between II and PIS are not linear discriminated, even at this temporal scale.

### Interspike interval variability differences between epileptiform-like activities

**Fig 3:**
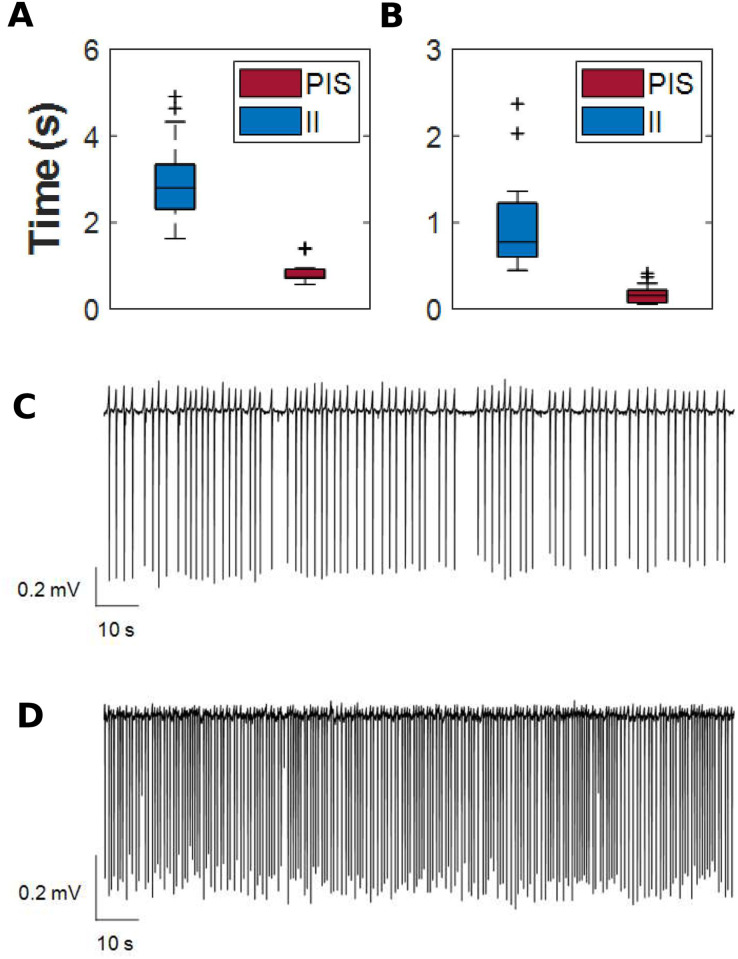
Interspike intervals for II and PIS pattern. (A) Interspike intervals (in seconds) distribution from all electrophysiological signals evaluated, independently of patient in which were recorded. The shaded region in gray represents the confidence limits for PIS epileptiform activity. Distributions of (B) means and (C) standard deviations of interspike intervals. The symbol “+” in all boxplots represents outlier values. Electrophysiological signals recorded in dentate gyrus of (D) interictal-like events and (E) periodic ictal spiking.

We calculated the interspike interval series from the electrophysiological recordings of the epileptiform-like activities, II and PIS, from all 12 human patients and all hippocampal subfields together (Fig A in S1 Text). In this analysis, statistical significant differences were found among their averages (H = 2935.55, *p* ≪ 0.0001). Comparing both epileptiform-like activities, regardless of the hippocampal subfields and patients, we found statistically significant differences between the means (W = 4.86, *p* ≪ 0.0001) and standard deviation (W = 4.86, *p* ≪ 0.0001) of interspike intervals for II and PIS (Fig 3A and 3B). Examples of electrophysiological records of II and PIS activities are shown in Fig 3C and 3D.

**Fig 4:**
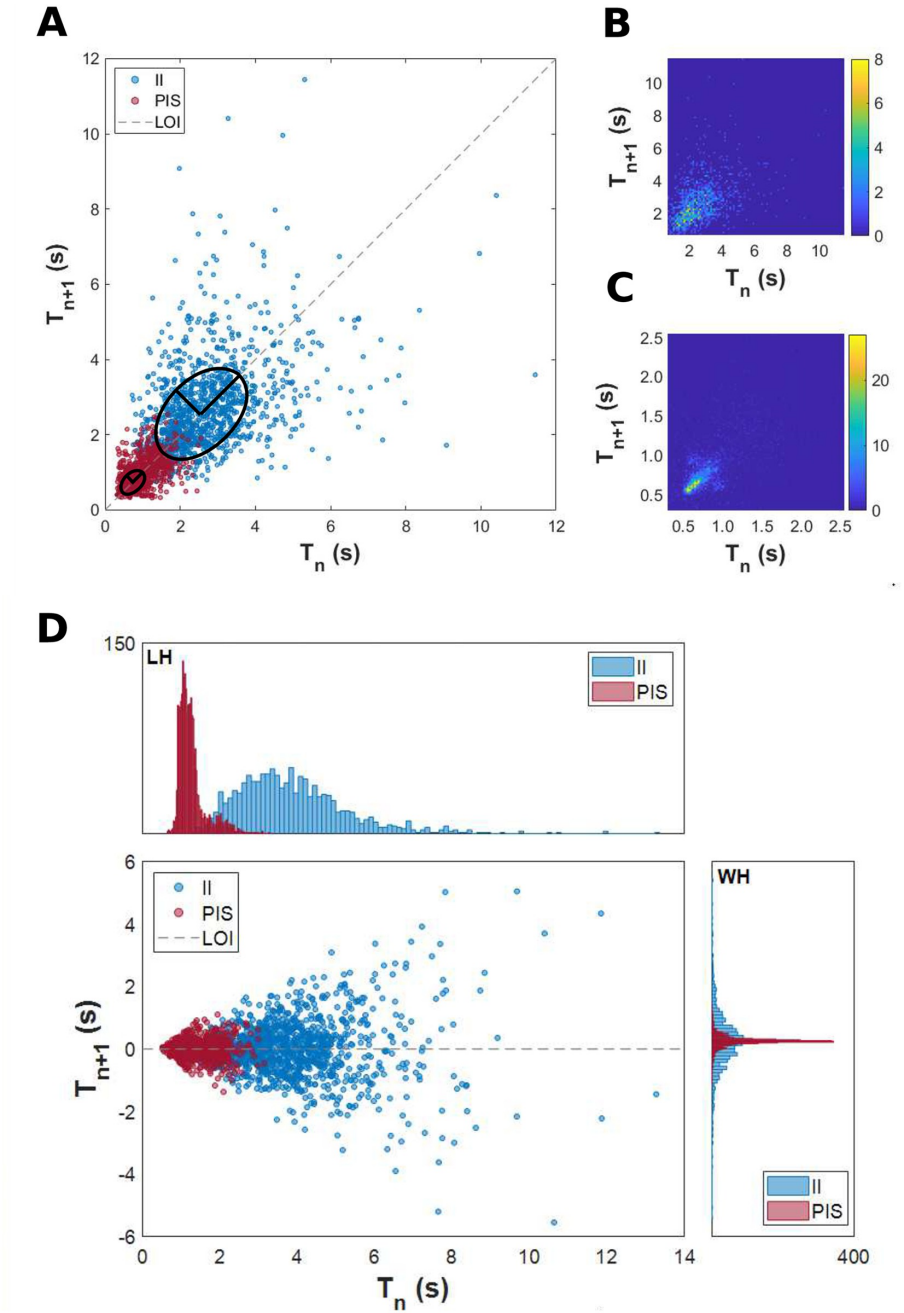
Spread pattern of interspike intervals. (A) Poincaré map from all interspike intervals. The II activity presents a higher variability of spread of its interspike intervals than the PIS pattern, with SD1II = 0.94 and SD2II = 1.48. The values of the minor axis and the major axis of the fitted ellipse for the PIS pattern are SD1PIS = 0.18 and SD2PIS = 0.35, respectively. Bivariate histogram of interspike intervals from (B) interictal-like events and (C) periodic ictal spiking. The color bar indicates the amplitude for each 100 bins. (D) “Length histogram” (superior panel) and “width histogram” (right panel) from Poincaré map points projected onto the line of identity and orthogonal to the line of identity, respectively. All histograms are defined with 100 bins.

By using Poincaré map, we were able to display dispersion patterns associated with the variability of the interspike intervals (Fig 4A) of II and PIS. Fig 4B and 4C exhibit the density spread patterns for these activities for both bivariate histograms, showing a higher concentration of cycle-to-cycle interspike intervals for PIS in contrast with a higher dispersion of interspike intervals for II activities. The parameters SD1 and SD2 also exhibit differences in magnitude; however, the ratio SD1/SD2 had the same difference for both epileptiform activities, II and PIS, indicating a higher continuous variation in both activities, since SD2 is higher than SD1.

Fig 4D shows the histograms of the Poincaré map associated with its projection onto the line of identity (LOI), yielding the “length histogram”, and perpendicular to the LOI, yielding the “width histogram”, for both epileptiform-like activities. The “width histogram” has standard deviation equal to SD1 and provides summary information on the short-term characteristics, whereas the “length histogram” has standard deviation equivalent to SD2 and carries the long-term information of time interval variability [48]. Both histograms, length and width, of the II activities and the length histogram of PIS activity presented positive skewness values (*γ* > 0), indicating a left concentration of points (Fig 4D). However, the “width histogram” of PIS activity presented negative skewness (*γ* < 0), indicating that the data are concentrated to the right side. Regarding the kurtosis, all histograms presented higher values than their associated normal distribution (*κ* > 3), as shown in Fig 4D. Nevertheless, the kurtosis from PIS histograms is higher than the kurtosis from II histograms (*κ*_*PIS*_ > *κ*_*II*_). It means that width and length histograms of PIS activity have their points concentrated around their mean, while II histograms have their points spread out and more uniformly distributed. This analysis shows that II activity presents higher variability in short and long-term time scale than PIS activity, and the deviations from the dispersion indices, SD1 < SD2, for both II and PIS, describe a gradual change of the event rate while maintaining a small variability of the consecutive event time intervals [74, 75].

**Fig 5:**
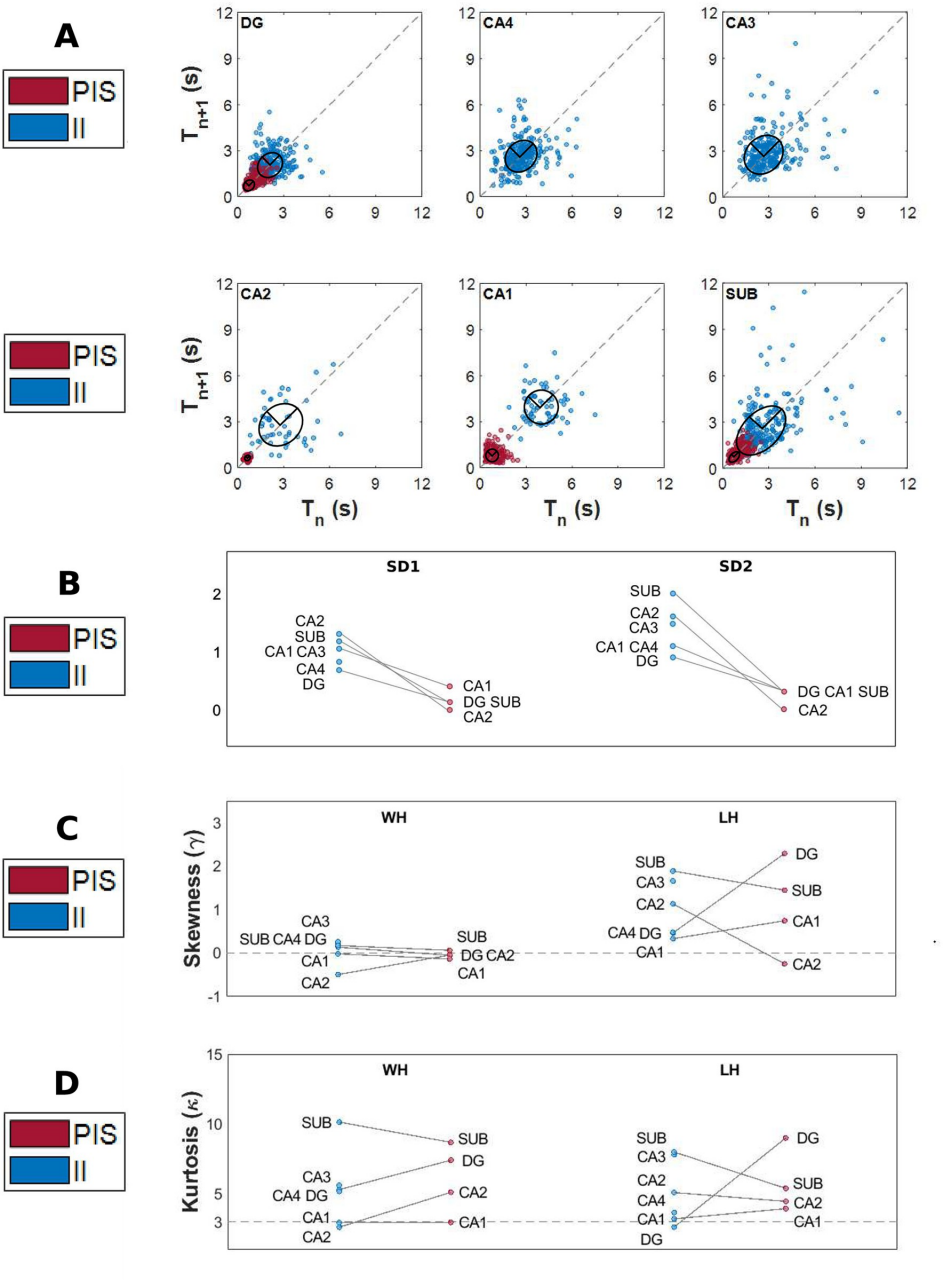
Spread pattern of interspike intervals into hipocampal subfields. (A) Poincaré map for each hippocampal subfield containing the interspike interval spread patterns. (B) Slope graphs of SD1 and SD2 values for each hippocampal subfield. (C) Slope graphs of skewness values of width (*γ*_*WH*_) and length (*γ*_*LH*_) histograms for each hippocampal subfield. (D) Slope graphs of kurtosis values of width (*κ*_*WH*_) and length (*κ*_*LH*_) histograms for each hippocampal subfield.

By comparing the variability of interspike intervals between each hippocampal subfield independently, we found that each one expresses particular spread patterns, as shown in Fig 5A. The standard deviations SD2 are higher than SD1 for both epileptiform activities, II and PIS, for all hippocampal subfields, except for PIS in the CA1 subfield (Fig 5B), indicating that this is the only hippocampal subfield that has a higher variance in instantaneous interspike intervals for this type of epileptiform-like activity. The values of the kurtosis and skewness were, respectively, positive and higher than three, for both epileptiform activities, in almost all hipocampal subfields, as shown in Fig 5C and 5D. The hippocampal subfields that presented skewness less than zero and kurtosis less than three with more frequency were CA1 and CA2. It is important to note that Poincaré map analysis showed a high robustness and accuracy even under high patient heterogeneities such as age, gender, initial precipitating injury, epilepsy duration, antiepileptic drugs, among others, highlighting the differences between II and PIS activities.

### Characterization of the underlying dynamics from epileptiform-like activities by recurrence factors

**Fig 6:**
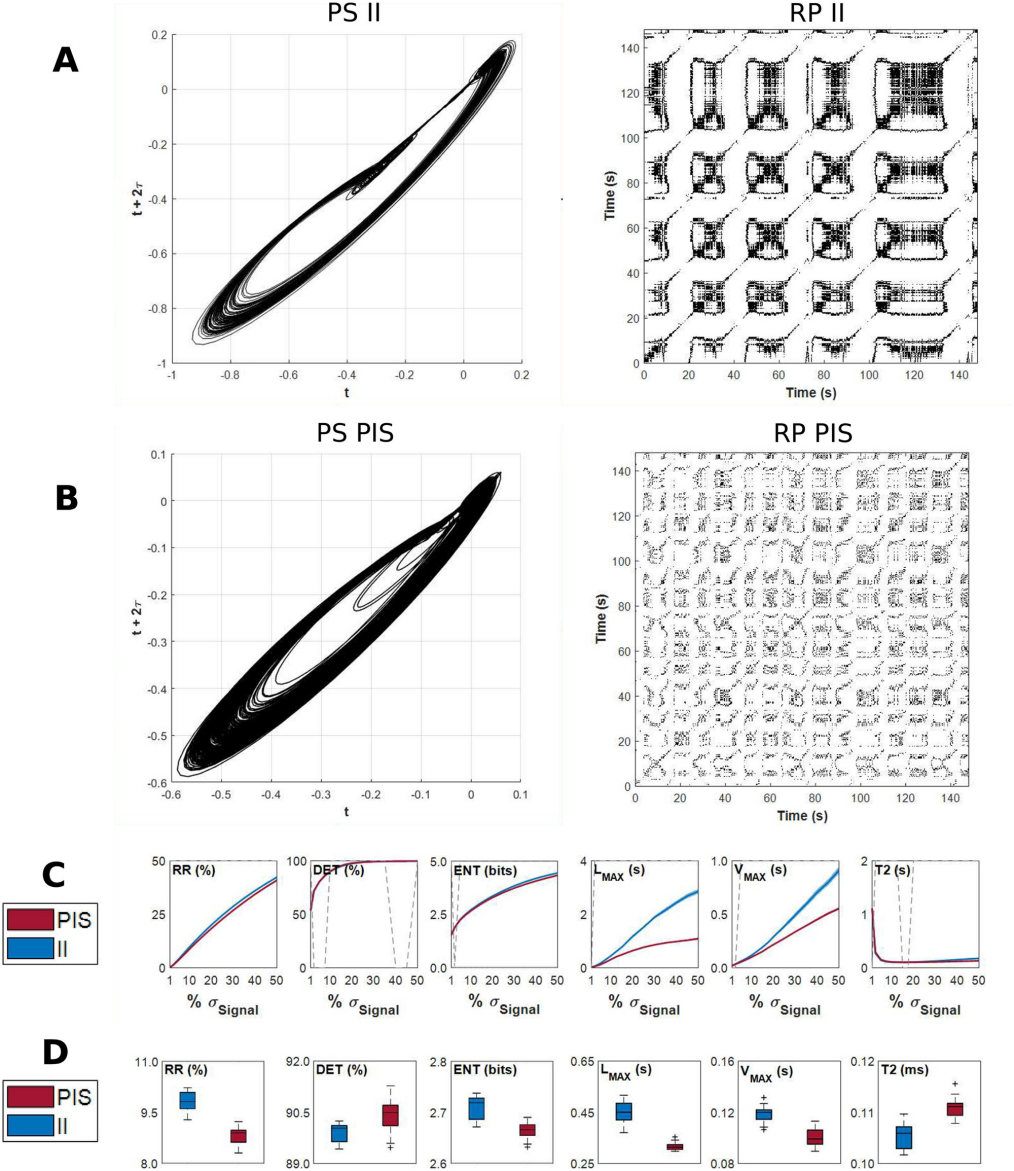
Dynamical characteristics of epileptiform-like activities in different time scales. Examples of phase space (left panel) and Recurrence Plots (right panel) of (A) interictal-like events and (B) periodic ictal spiking epileptiform activity, from time series recorded on dentate gyrus. (C) RQA factors in function of the recurrence threshold (*ϵ*) parameter. The parameter *ϵ* used to calculate Recurrence Plots was variated from 1% to 50% of the standard deviation of each electrophysiological record, with steps of 2.5%. The six RQA factors allow to compare II and PIS activities according to their levels of chaoticity, stochasticity and determinism. The dotted gray line indicates the regions where the RQA factors are statistically different (*p* < *α*) among both epileptiform activities, II and PIS, using Wilcoxon test (0’s means *p* > *α*). (D) Distribution of RQA factors considering recurrence threshold as 10% of the standard deviation of each electrophysiological record. Each point of the boxplot was calculated into RPs constructed from windows of 10 s of the electrophysiological data analyzed (as RPs shown in (A) and (B)).

All RQA factors presented significant differences between II and PIS epileptiform-like activities (Fig 6C and 6D), showing that these activities are distinguished by nonlinear features. Through this analysis, we found that the II activity presented a higher global recurrence than the PIS activity, measured by the recurrence rate (RR) factor. This means that the probability that any state of the system will recur is higher for II than PIS [37, 53]. In addition, PIS patterns presented more white bands, indicating abrupt changes in the dynamics of the system [60]. These white bands suggest that a set of dynamic conditions or states are not visited, or they do not occur [53, 60], in other words, suggesting loss into access to some dynamic physiological conditions [76].

The maximal diagonal line length (*L*_*max*_) factor was higher for II than PIS, indicating that II activities present periods of event-recurrences on the phase space. Although higher values for *L*_*max*_ indicate a less stochastic dynamics, with less divergent trajectories in the phase space, we have also to consider its variance. Since the *L*_*max*_ variance of II activities are higher than PIS activity, this means that the system dynamics oscillates between periods with high recurrences and periods with low recurrence, indicating the occurrence of regular epochs with fast events, like bursts.

The maximal vertical line length (*V*_*max*_) factor is higher for II than PIS, indicating regularity of the time series in relation to its noise. This means that the II activity is more irregular in long-term time scale, presenting small changes in the entire recording. Regarding recurrence times of the second type (T2), this factor is higher for PIS than II, indicating that PIS is a more periodic activity and remains around a given event longer than II. All these results suggest that the II activity reflects a more stochastic and chaotic dynamics, whereas the PIS activity is related to a dynamical system of a more deterministic nature.

**Fig 7:**
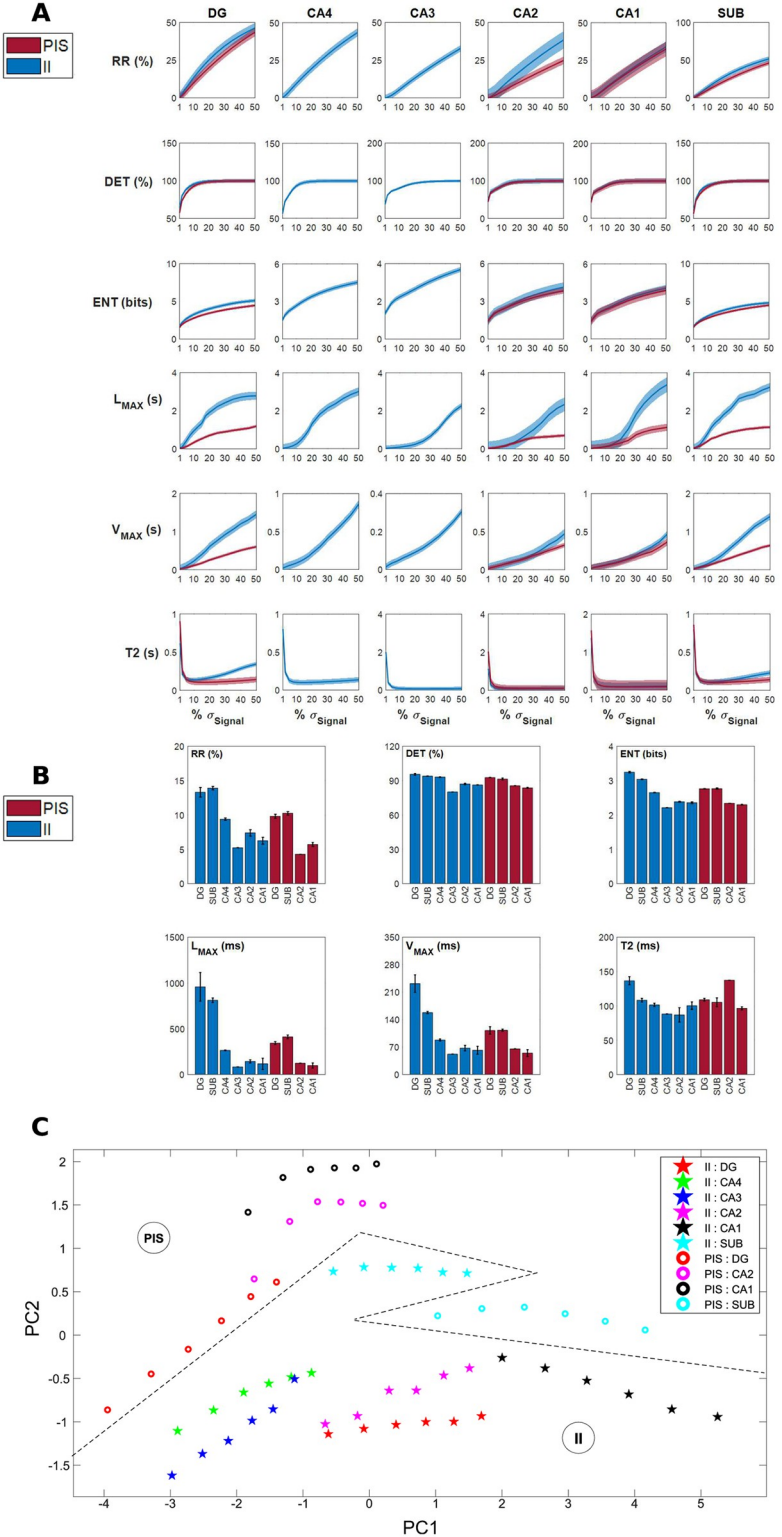
Dynamical particularities of II and PIS activities of each hippocampal subfield. (A) RQA factors in function of the recurrence threshold (*ϵ*) for each hippocampal subfield. The line represents the mean value and the shaded region represents the confidence interval of the mean. (B) Mean values of the RQA factors in each hippocampal subfield for a recurrence threshold of 10% of each electrophysiological recording standard deviation. Error bars indicate SEM. (C) PCA analysis applying *ϵ* of 10% to 15% of each electrophysiological recording standard deviation.

Furthermore, to highlight the particularities of II and PIS for each hippocampal subfield, we applied RQA to the electrophysiological signals recorded independently in each of these HP regions. Fig 7A shows the RQA factors values in relation to the recurrence threshold (*ϵ*) for each hippocampal subfield, considering both types of epileptiform-like activity, II and PIS. This analysis showed that each subfield presents a particular dependence in *ϵ*. Since *ϵ* reflects the resolution based on which we identify optimally the RQA factors associated with each electrophysiological record, it means that each subfield expresses II and PIS activities in a particular way. This autonomous activity dependent of *ϵ* can also be seen in Fig 7C, where we applied a PCA keeping five values of *ϵ*, for each activity in each hippocampal subfield, and used the six RQA factors of PCA main features. In this way, by using RQA factors, in this *ϵ* optimal range, we were able to entirely classify all subfields according to II and PIS activity, highlighting their individual particularities and group similarities.

Considering the recurrence threshold fixed at 10% of the standard deviation of each electrophysiological recording, our results indicate that the dentate gyrus and subiculum are the hippocampal subfields with higher recurrence rate (*RR*), Shannon entropy (*ENT*), maximal diagonal line length (*L*_*max*_) and maximal vertical line length (*V*_*max*_) in both epileptiform-like activities, II and PIS (as shown in Fig 7A and 7B). It means that the dentate gyrus and subiculum perform a more irregular and complex epileptiform activity. These two hippocampal subfields present a dynamic with more stochasticity and chaoticity than the hippocampus proper (CA1-CA4), indicating the activity from these regions are mainly expressed by a coupled and complex system. Furthermore, CA2 presented higher determinism (*DET*), followed by a recurrence rate (*RR*) lower than 5%, with high recurrence times of the second type (*T*2) for the PIS activity, which suggests this region has a more regular dynamics than the other hippocampal subfields.

### Characterization of the underlying dynamics from computational models by recurrence factors

**Fig 8:**
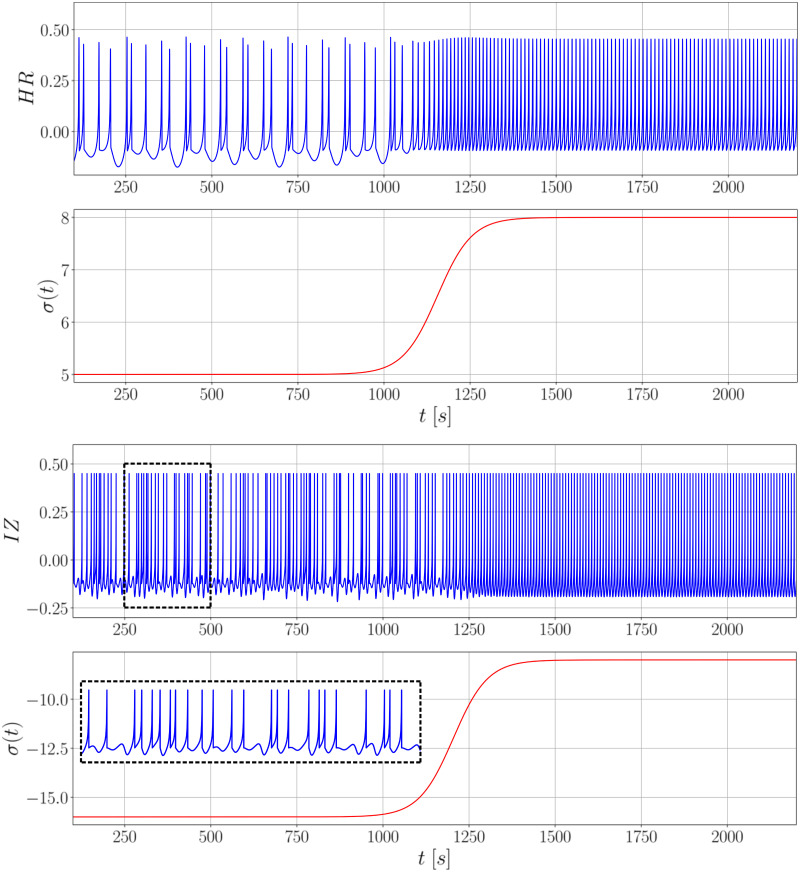
Transition between chaotic to regular behavior for the neuron dynamic models. The transitions for the Hindmarsh-Rose model (above) and Izhikevich model (below) are based on a sigmoid wave in function of the control parameters *I* and *d*, respectively. The time windows of both responses are similar, although Izhikevich’s model evolves in a relatively faster time scale.

Fig 8 presents the transition effect from a chaotic spiking behavior (similar to II patterns) to a regular spiking behavior (similar to PIS patterns). This is attained by linking the state variables of both systems to a sigmoid wave (or step wave) in terms of control parameters (further details can be found in S1 Text). Both models capture the effect of double spiking during the chaotic regime, where the irregular interspike intervals are highlighted, whereas regular intervals are present in the constant spiking period.

**Fig 9:**
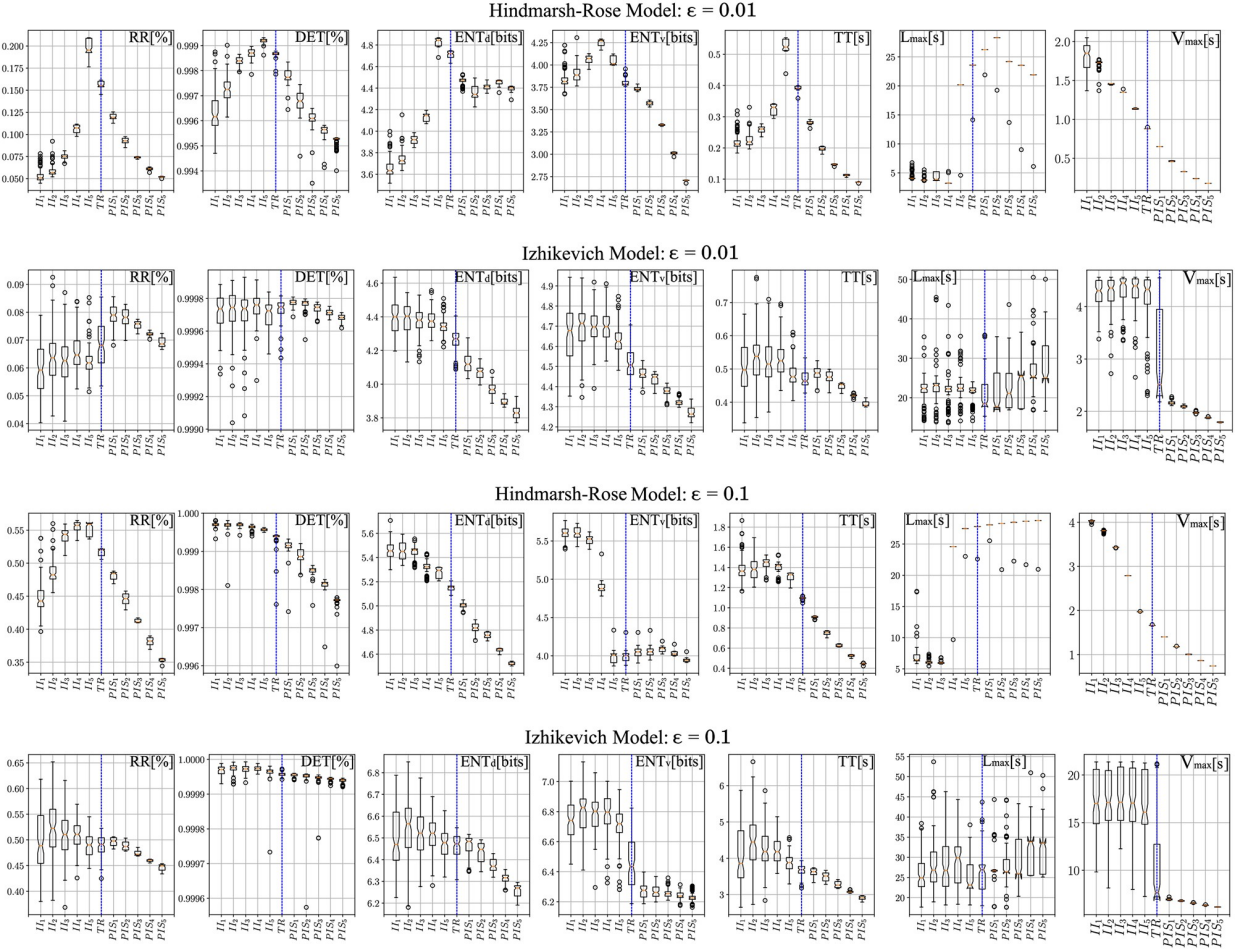
Effect of threshold on the RQA parameters considering the Hindmarsh-Rose and Izhikevich models. Box plots for the six parameters studied in this work are presented (an exception is the vertical entropy ENTv, which is a complementary result) for the 11 cases and 2 different threshold values (*ϵ* = 0.01, 0.1): 5 for interictal activity (II), 1 transition (TR) and 5 for the ictal activity (PIS). Vertical blue dashed lines indicate a visual separation between the chaotic and regular spiking.

Fig 9 presents the boxplots for all RQA parameters with the 11 epochs considered (*II*_1_, *II*_2_, *II*_3_, *II*_4_, *II*_5_, *TR*, *PIS*_1_, *PIS*_2_, *PIS*_3_, *PIS*_4_, *PIS*_5_, where *TR* is a transition), for different thresholds. We assumed the following thresholds *ϵ* = 0.01, 0.025, 0.05, 0.10, to keep the recurrence percentages close to the real ones (as seen in Figs 6 and 7). Complementary results for *ϵ* = 0.025 and 0.05 can be found in Fig B in S1 Text. From these two figures, where all RQA factors are compared for all HP subfields, the overall statistical quantification is that all values from II are higher than those from PIS, considering *RR*, *ENT*, *V*_*max*_ and *L*_*max*_. Additionally, the following effects are observed as the threshold is increased in the computational models.

The Recurrence (*RR*) factor in the HR model, although it presents extreme situations during chaotic and regular regimes, it tends to decrease as it moves farther from the transition value (*TR*). A similar effect is seen in the IZ model, but with a larger variance. The Determinism (*DET*) factor is consistent with the real signals too, showing that a high determinism is present in both chaotic and regular conditions. The Entropy (*ENT*) factor during the chaotic regime is clearly higher than the regular spiking regime in both models. Indicating that chaotic behaviors present more complex activities. The Trapping time (*TT*) and maximum vertical lines (*V*_*max*_) factors are closely related and present higher values for the chaotic periods in both models. Showing that chaotic regimes tend to revisit similar sates more often, unlike regular activities, which resemble arbitrarily long oscillations. The longest diagonal line (*L*_*max*_) factor in the HR model presents well-defined transitions between states in comparison with the IZ model.

**Fig 10:**
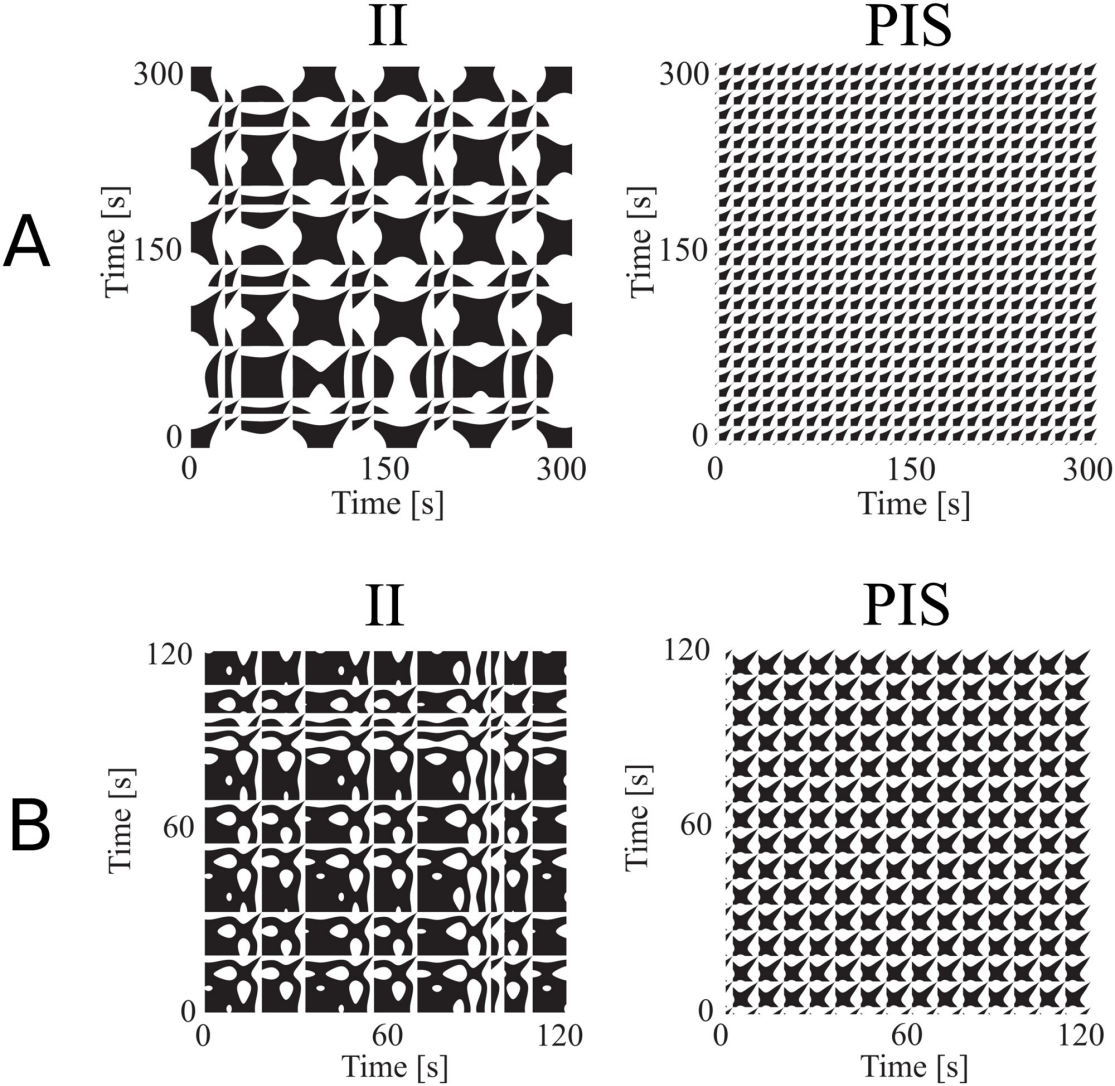
Recurrence plots for the simulated neuron dynamic models. A) Hindmarsh-Rose model considering a time window which represents a 1:3 firing ratio; B) Recurrence plots for the Izhikevich model considering a time window which represents a 2:3 firing ratio. Note that the II pattern presents larger but less frequent repeating structures, whereas PIS presents smaller but more frequent ones, as seen in Fig 6.

At last, Fig 10 presents selected recurrence plots for both dynamic models. Although their time scales are inherently different, the ratios of 1:3 and 2:3 were kept for the spiking patterns of the HR and IZ models, respectively, to represent the real event rates (*r*) of *r* < 40/*min* for II, and *r* > 40/*min*, for PIS [7, 8].

Through these analyses we see that IZ and HR models are complementary, where each one captures better a specific regime related to the epileptic activities found along the electrophysiological recordings. While the Hindmarsh-Rose model captures better the repeating plaid pattern, the Izhikevich model captures better the stingray-like structures with big white gaps inside, present in real signals (Fig 6). Finally, for both models the II regime presents fewer but larger structures, whereas the PIS region presents more but smaller ones (as shown in Fig 6).

## Discussion

This work investigated nonlinear dynamics related to electrophysiological patterns from the two most prevalent epileptiform-like activities in slices of human sclerotic hippocampus from 12 patients with pharmacoresistant TLE. To quantify the nonlinear features related to these signals we applied the Poincaré Map and Recurrence Quantification Analysis (RQA) to the interictal-like events II and periodic ictal spiking PIS epileptiform activities, characterizing them according to their stochasticity, determinism and chaoticity dynamics. Through these analyzes we also identified specific patterns associated with each hippocampal subfield, suggesting that II and PIS are not only distinct electrophysiological expressions with different nonlinear dynamics but also that each hippocampal subfield has its own way to manifest these two epileptic activities.

Poincaré Maps showed that interspike intervals of both epileptic activities, II and PIS, contain information and can totally distinguish them. They indicate that the variability of interspikes is important and the way epileptic spikes are discharged in time must also be considered. By evaluating other statistical parameters, such as skewness and kurtosis, we also found that each hippocampal subfield expresses II and PIS in a distinct way. Additionally, from the RQA analysis we showed that PIS patterns are much less stochastic and less chaotic than II, exhibiting great self-similarity and temporal regularity in their waveforms and in their interspike interval variability, while II patterns were shown to be more stochastic and at the same time more chaotic in their waveforms, interspike intervals and amplitude.

Linear techniques, such as cross-correlation and PCA, were not able to properly distinguish II from PIS activities. The differences between II and PIS were only highlighted when the cross-correlation of the interspike intervals of both activities were evaluated (as shown in Fig 2A and 2B). These analyzes emphasized the self-similarity of PIS interspike intervals and the low self-similarity of interspike intervals of II activity. This finding shows that, besides the different event rates, the way these epileptiform events vary over time also plays an important role, suggesting that II and PIS are expressions of two distinct mechanisms (Fig 4A).

Analogous to heart rate variability investigations, where the Poincaré map is used to characterize different cardiac and autonomic conditions [46, 77–82], we found periods of acceleration- and deceleration-like effects, observed by high spreading cluster patterns related mainly to II activities [74, 75, 83]. Since any acceleration- and deceleration-like effects are provided by some systemic and endogenous influence [74, 75, 84], it indicates that II dynamics are probably yielded by some coupling interaction. These effects can be seen also in Fig 4 (and Fig A in S1 Text), where periods of decreasing size and increasing size of interspikes appear periodically.

From the phase-space and Recurrent Plots (RPs) of II and PIS activities (Fig 6A and 6B), it is possible to see how these activities are statistically different and how II patterns exhibit more chaotic features than PIS patterns [37, 56]. In general, periodic signals present longer diagonal lines in their RPs, while chaotic signals present shorter diagonal lines, and stochastic signals present no diagonal lines (or short spurious ones). In this way, the quantification of RPs shows that PIS activity presented a lower RR factor but a higher *DET* factor than the II activity, indicating that PIS is more periodic with more regular and predictable dynamics. The factors *V*_*max*_ and *T*2 show that the PIS activity presented less variance along their trajectories, point-to-point. Since *V*_*max*_ essentially relates the presence of singular states in which the system is temporally stuck in a specific pattern and *T*2 is the time that a point takes to reoccur, on average, we can also interpret that PIS is more stationary along time than II activity (Fig 6D). The major variance found in *L*_*max*_ for II activity indicates the occurrence of divergent periods with many recurrences interspersed by periods with few recurrences, pointing once again to periods of acceleration and deceleration [13]. The factor *L*_*max*_ can be also associated with a positive Lyapunov exponent which is indicative of chaoticity [13]. This is also emphasized by the *ENT* factor, which measures the Shannon entropy of diagonal line length distributions. It was higher for II than PIS, indicating that II is a more complex activity than PIS.

Taking these recurrence factors together we can conclude that the II activity exhibits many chaotic features, especially when we consider the temporal evolution of events, like interspike intervals, and its amplitude variance. However, on a shorter time scale, II presents more complex and stochastic behavior than PIS patterns. PIS activity expresses more deterministic and regular dynamics related to its interspike event distribution over time, and less amplitude variation associated with its signal morphology. In terms of an oscillatory physical interpretation, II activity is more similar to driven-dumped oscillations while PIS is more similar to harmonic oscillations [58, 85, 86].

From these nonlinear analyzes it was also possible to classify the human hippocampal subfields according to their own epileptiform activity dynamics. Stochastic and chaotic dynamics were mostly found in the dentate gyrus (DG) and subiculum, while a more deterministic dynamic of the epileptiform-like activity was mainly encountered in CA1-CA4. Anatomically, the hippocampus usually obeys a unidirectional and laminar pattern where the DG covers mainly CA3 and CA4 regions, which in our analyzes were the regions that did not present any PIS activity (Fig 5) [87, 88].

The DG and subiculum present similar Poincaré Maps, but looking at their RQA factors, the DG exhibits higher values for *L*_*max*_, *ENT*, *V*_*max*_ than the subiculum, which indicates chaotic effects. Since the DG is the main input of the hippocampus and the subiculum is the main output [89], the RQA factors indicate that the activity from the subiculum is more stochastic but highly complex. This result could help to understand how the information is disrupted in the hippocampus during epilepsy, mainly because the subiculum has been described as playing an important role in the initiation and maintenance of epileptic discharges in temporal lobe epilepsy [90]. Both DG and subiculum, in comparison with the other structures, CA1-CA4, exhibit the most complex patterns of II and PIS. Fig 6C for example, shows how all structures are completely distinguished through their RQA factors in the PCA space, even when six threshold values are considered. It indicates that not only the patterns II and PIS are totally different, but they are also expressed in a different and autonomous way in each hippocampus subfield. In particular, we can see how PIS and II activities are well separated from the DG while the same patterns from the subiculum are quite close in the PCA space.

The RQA factors calculated from the computational models reinforced the idea that II and PIS are transient activities expressed by modulations of parameters in the IZ and HR models. They also showed the same tendency found in the electrophysiological recorded patterns, for a higher global recurrence in II patterns in comparison to PIS. Additionally, the higher entropy for simulated II, than simulated PIS, indicates that interictal activities are more complex than ictal activities. Although some RQA factors had different behaviors between IZ and HR models, when assessing the whole set of factors, they converged in the same type of phenomenon description.

The most important contribution of these simulations is the clear possibility to convert one type of activity to the other by changing internal parameters associated with environment and physiological conditions. By modulating the sigmoid function, for instance, used to model the smooth transition between II and PIS, it helped explain how neurons synchronize over time, leading to a seizure. It is worth mentioning that the opposite direction, from regular activities to more complex ones, can also be obtained by inverting the order of the parameters used during the simulations. Since such transitions imply in a change in the event rates (*r*), they also reflected the effect of acceleration and deceleration verified in the real signals.

In terms of transition between the interictal and ictal conditions, there is not a general mechanism capable of explaining it. As discussed in [91, 92], there are computational models able to generate such transitions, but based on distinct mechanisms: external excitations which modulate the system’s parameters (also known as autonomous systems), bistable or multistable models which present the so-called bifurcations (semi-autonomous), and intermittent models (or autonomous models), which are inherently unstable.

The HR and IZ models present a considerable rich spectrum of neural spiking [40, 41]. Both have configurations that comprise rest and firing states, whose transition is modeled by bifurcations [71, 72]. Furthermore, the type of spiking can also be modulated by varying the parameters of the models, thus presenting time signatures with regular or irregular interspike intervals, one characteristic of chaotic systems. Thus, these models present features of non-autonomous and semi-autonomous systems.

Another relevant factor directly involved in the anticipation or unpredictability of the transition are internal fluctuations, or noise [92]. In fact, the transition caused by such fluctuations (as well as the time spent between pre-ictal and ictal states) can even be modeled considering a probabilistic approach [93]. In the development of the Epileptor model, which was partially inspired by the HR model, it was verified that the addition of noise to the model can, indeed, bring forward a seizure, and experimental tests endorsed this observation [73].

In this sense, in fact, the gradual increase/decrease of the parameter magnitudes involved in the models, IZ and HR, is only one of the possible several reasons that may cause the transition between interictal and ictal states [91]. However, the main purpose is to verify that the qualitative signal differences can be (theoretically) approximated by the mathematical models, as well as quantified using nonlinear techniques (RQA) by their time signatures, especially in terms of interspike intervals. Some of the mechanisms involved in the transitions related to the computational models presented here are briefly covered in S1 Text, and further information about these transitions can be found in [71, 72].

Furthermore, by taking samples considering specific control parameters in the sigmoid function, and varying the RQA threshold, different configurations of the same system were obtained. It means that, depending on the degree of neural synchronization, we are able to represent the spiking patterns and the differences in terms of RQA factors and time signatures, as verified for the HP subfields. S1 Text provides a summary of the biophysical meanings associated to the parameters of each model that helps to understand their rule in these scenarios.

Our results also introduced new questions that deserve to be discussed. Why does an epileptic hippocampus prevalently produce two different patterns, like II and PIS activities? What mechanisms would be yielding these two distinct activities? Why does each HP subfield express each pattern differently, but consistently? Can we interpret it as an integrated but autonomous processing, or is it only spurious environmental effects? Is it a natural physiological processing, or does it reflect a particular manifestation of epilepsy?

The application of new tools and concepts related to nonlinear dynamics motivates the formulation of some possible explanatory conjectures to instigate future investigations:

### Biological disruption of neural circuits due to sclerosis

All the results and interpretations presented here are based on the premise that temporal lobe epilepsy is directly associated with hippocampal sclerosis [1, 4, 5], which is histologically characterized by neuronal death, gliosis, aberrant neural paths, among others. They promote erratic structural and functional changes in each hippocampal subfield over time [2, 3, 94, 95] and disrupt the neural homeostasis, which may in turn result in a network reorganization. Therefore, the hippocampal sclerosis could be interpreted as an intrinsic seizure-susceptible medium that, in time, can express specific recurrent dynamics, such as II and PIS and all their subtle transitions marked by different RQA-*ϵ* ranges.

The basic synaptic circuit of the hippocampus goes from CA1 to the subiculum and it leaves the hippocampus to the entorhinal cortex. The predominance of Glutamatergic pathways predisposes the hippocampal circuit to reverberant activities conducive to epileptiform patterns [90]. Together with a possible mechanism of plasticity induced by these patterns, which retrograde the subiculum to CA1, it may produce reverberant connections that increase its hyperexcitability. This mechanism is one of the main explanations for the emergence of epileptiform patterns in TLE. From our results, it is clear that there is a particular activity in the CA1-subiculum and in the DG. Additionally, the homogeneity and absence of any special synaptic path between CA3 and CA4 is also expressed in our analysis (Fig 5). The particular patterns found in CA2 are also corroborated by the literature, which has reported CA2 as a modulatory function in HP circuits that is not well understood [96], mainly concerning the activity between CA1 and CA3. We cannot guarantee that a CA1-subiculum shortcut can, in some way, spread out the epileptiform activities along HP proper and DG only through our results, but surely they show that the role of all other subfields should be better investigated in the epileptogenic process.

### Disruption of functional networks and homeostatic imbalance between excitation and inhibition

The hyperexcitability promoted by the experimental approach used to (re)induce the epileptiform activities was able to generate two different types of epileptiform activities, II and PIS, that never occur concomitantly in the same subfield of the sclerotic tissue [28], suggesting a possible difference in the underlying network configurations. Therefore, the epileptic patterns are directly associated with a synaptic homeostasis imbalance through an increase of excitatory neurotransmissions as well as a decrease of inhibitory neurotransmissions, yielding neuronal hyperexcitability and synchronization [97–100].

The activity of some classes of subicular and CA1 cells can change dramatically under certain environmental conditions, such as the application of potassium channel blockers [90]. These neurons are glutamatergic, and although the exact control mechanisms promoted by GABAergic inhibitors still need to be clarified, any change in the biochemical mechanisms that balance these networks can trigger seizure patterns [101]. In this way, since we know that the signal patterns analyzed here, II and PIS, were recorded from sclerotic tissues under a bicuculine induction, which inhibits GABAergic pathways and substantially increases the environmental excitability, we can ask whether it would be enough to make II be different from PIS patterns in all aspects we evaluated. Or does it only exacerbate a specific and latent activity provided by a sclerotic circuit? Our interpretation is that, assuming the excitatory induction was homogeneous and similar for all tissue and all samples, the dynamic differences of II and PIS occurred either due to some structural configuration (such as gliosis, neuronal death, aberrant networks), as discussed above, or functional activity (inversion of GABAergic potentials, homeostasis breaking) in the neural circuits of each subfield.

Furthermore, the stochasticity and chaoticity predominance in II activity could be conjectured as an expression of a partial failure of an inhibitory mechanism. The system erratically “tries” to inhibit neural hyperexcitations but fails. On the other hand, since recordings of PIS exhibit a more deterministic activity, it expresses a pattern characterized by strong failure of its inhibitory mechanisms. The system cannot inhibit the neural hyperexcitations properly, and without proper inhibition the neural system discharges freely and more regularly. Therefore, an optimal inhibitory process would express no epileptiform activity, reestablishing the neural homeostasis and hence PIS activities would be a functional precursor of generalized seizures. Unfortunately, we can only classify the activities according to their dynamics that indicate the type of underlying physical system.

In this way, the lack of PIS activity in the CA3 and CA4 subfields could be an indicative that these regions present higher inhibitory mechanisms. Previous reports also showed that the CA3 subfield of rats was resistant to generating seizure-like events in an in vitro model using high extracellular potassium [102]. Furthermore, findings obtained in brain slices of pilocarpine-epileptic rats, showed that the CA3 subfields rarely exhibited spontaneous bursts [103]. Regarding the CA4 hippocampal subfield, the analysis is more complex, since the CA4 presents distinctive histological features in the sclerotic hippocampus, such as scattered hypertrophic neurons, granule cell dispersion, and expressive neuronal loss [2]. Previous studies have shown that even with a severe neuronal loss, neuronal networks with few cells are able to sustain epileptiform activity [104, 105]. In addition, the CA4 subfield exhibits a diversity of neuron types [106], including parvalbumin-interneurons, which are predominantly inhibitory cells. Therefore, even few parvalbumin-interneurons could still inhibit the activity of mossy cells, decreasing seizure susceptibility in the CA4 subfield.

In summary, this work showed for the first time that there is distinct information associated with II and PIS epileptiform-like activity hidden in nonlinear dynamics beyond their a priori rate differences. In particular, by using the Poincaré map and RQA we were able to categorize these recordings according to their levels of stochasticity, chaoticity and determinism. The II activities were shown to be more chaotic with complex rhythmicity than PIS activities. The nonlinear dynamic differences found between II and PIS lead us to conjecture that they are expressions of different seizure susceptibility, probably due to different epileptogenic stages. We also identified that each hippocampal subfield expresses II and PIS activities in a specific and different way, which can be interpreted as consequence of the specific neuronal circuitry associated with each subfield that responds differently to the epileptogenic process. In this way, we believe this study opens a new door for an investigation on the characterization of the causal mechanisms underlying the different inhibitory aspects of interictal-like events and periodic ictal spiking in TLE. A limitation of this study is the lack of an appropriate control tissue, but using the two computational models, IZ and HR, we were able to guide our interpretations associated with the dynamic transitions of II and PIS activities.

## Supporting information

S1 Text Complementary results including real epileptiform analysis and both Hindmarsh-Rose and Izhikevich models. This appendix is divided into six main sections: Identifying dynamic structures in real signals; Adapting neuron dynamic models to the epileptiform activity; Applying RQA to the dynamic models; Analysis of RQA parameters for both models; Biophysical meanings associated to the parameters of each model; Adherence of the Computational Models to the Electrophysiological Recordings. **Fig A. Main structures found in the hippocampal subfields during the interictal (II) and ictal (PIS) states**. Note that a more irregular and less frequent spiking pattern is observed for all selected epochs from II, whereas a more regular and frequent spiking pattern is observed for all selected epochs from PIS. All signals were properly filtered to reduce the effect of background or unrelated activity. A time window of 50 seconds is considered for all cases for purposes of visual comparison. **Fig B. Box plots for the six parameters studied in this work (an exception is the vertical entropy, which is a complementery result) for the 11 cases considering the Hindmarsh-Rosel and Izhikevich models and 2 different treshold values (*ε* = 0.025, 0.05): 5 for interictal activity (II), 1 transition (TR) and 5 for the ictal activity (PIS)**. Vertical blue dashed lines indicate a visual separation between the chaotic and regular spiking. **Fig C. Logistic regression to classify the computational models in II and PIS conditions based on the AR coefficients obtained from real signals**. The first picture (left above: II signals of HR model) indicates in blue that the artificial coefficients labeled as II activities were correctly classified as II patterns, whereas orange color indicates that the artificial coefficients from II activities were incorrectly classified as PIS patterns. The second picture (left below: PIS signals for HR model) indicates in orange that the artificial coefficients labeled as PIS activities were correctly classified as PIS patterns, whereas blue color indicates that the artificial coefficients from PIS activities were incorrectly classified as II patterns. The pictures showed right above and right below represent the same analysis for the IZ model. The curves are plotted considering their probability of correct classification related to their confidence intervals at 95% confidence, for > 50 samples. Noise varies from 0% to 10% in 20 steps of 0.05% each. **Fig D. Root Mean Square Error (RMSE) between combinations of real II/PIS activities and their reconstructions using II/PIS coefficients (confidence intervals)**. The titles indicate which group of real signals are compared (II or PIS). The legends indicate which types of coefficients were used to reconstruct them and the respective computational models. Blue dots indicate reconstructions using II coefficients; red squares indicate reconstructions using PIS coefficients. The curves are plotted considering their confidence intervals at 95% confidence. Noise varies from 0% to 10% in 20 steps of 0.05% each. **Fig E. Root Mean Square Error (RMSE) between combinations of real II/PIS activities and their reconstructions using II/PIS coefficients (boxplots)**. The titles indicate which group of real signals are compared (II or PIS). The curves are plotted considering boxplots in a log scale: to the left, boxplots of reconstructions using II coefficients; to the right, the same but for PIS coefficients. Noise varies from 0% to 10% in 20 steps of 0.05% each.
(PDF)
