# Supplementary material for: Chaotic and stochastic dynamics of epileptiform-like activities in sclerotic hippocampus resected from patients with pharmacoresistant epilepsy
Source: PLoS Comput Biol. 2022 Apr 13;18(4):e1010027. doi: 10.1371/journal.pcbi.1010027 (PMC9037954; doi:10.1371/journal.pcbi.1010027)
Supplement: S1 Text — This appendix is divided into six main sections: Identifying dynamic structures in real signals; Adapting neuron dynamic models to the epileptiform activity; Applying RQA to the dynamic models; Analysis of RQA parameters for both models; Biophysical meanings associated to the parameters of each model; Adherence of the Computational Models to the Electrophysiological Recordings. Fig A. Main structures found in the hippocampal subfields during the interictal (II) and ictal (PIS) states. Note that a more irregular and less frequent spiking pattern is observed for all selected epochs from II, whereas a more regular and frequent spiking pattern is observed for all selected epochs from PIS. All signals were properly filtered to reduce the effect of background or unrelated activity. A time window of 50 seconds is considered for all cases for purposes of visual comparison. Fig B. Box plots for the six parameters studied in this work (an exception is the vertical entropy, which is a complementery result) for the 11 cases considering the Hindmarsh-Rosel and Izhikevich models and 2 different treshold values (ε = 0.025, 0.05): 5 for interictal activity (II), 1 transition (TR) and 5 for the ictal activity (PIS). Vertical blue dashed lines indicate a visual separation between the chaotic and regular spiking. Fig C. Logistic regression to classify the computational models in II and PIS conditions based on the AR coefficients obtained from real signals. The first picture (left above: II signals of HR model) indicates in blue that the artificial coefficients labeled as II activities were correctly classified as II patterns, whereas orange color indicates that the artificial coefficients from II activities were incorrectly classified as PIS patterns. The second picture (left below: PIS signals for HR model) indicates in orange that the artificial coefficients labeled as PIS activities were correctly classified as PIS patterns, whereas blue color indicates that the artificial coeffici [file pcbi.1010027.s001.pdf]

Chaotic and Stochastic Dynamics of Epileptiform-Like Activities in Sclerotic Hippocampus Resected from Patients with Pharmacoresistant Epilepsy

Noemi S. Araújo, Selvin Z. Reyes-Garcia, João A. F. Brogin
Douglas D. Bueno, Esper A. Cavalheiro, Carla A. Scorza, Jean Faber

Identifying Dynamic Structures in Real Signals

Fig A presents selected recordings from the hippocampal subfields in the two conditions: interictal and ictal activities. Although in different ranges, a same window of 50 s was used for each one to emphasize the difference in event rates. Note that important structures can be identified: i) for the interictal state, the interspike intervals are less regular; also, depolarizing after-potentials and multiple spikes at once are clearly observed; ii) for the ictal state, in turn, the interspike intervals are more regular and frequent, that is, the number of spikes per unit of time is higher. Other background, more complex activities are naturally involved in each of them; however, as a general observation via a thorough visual inspection, these features are considered the most relevant ones, as explained and endorsed in the next section.

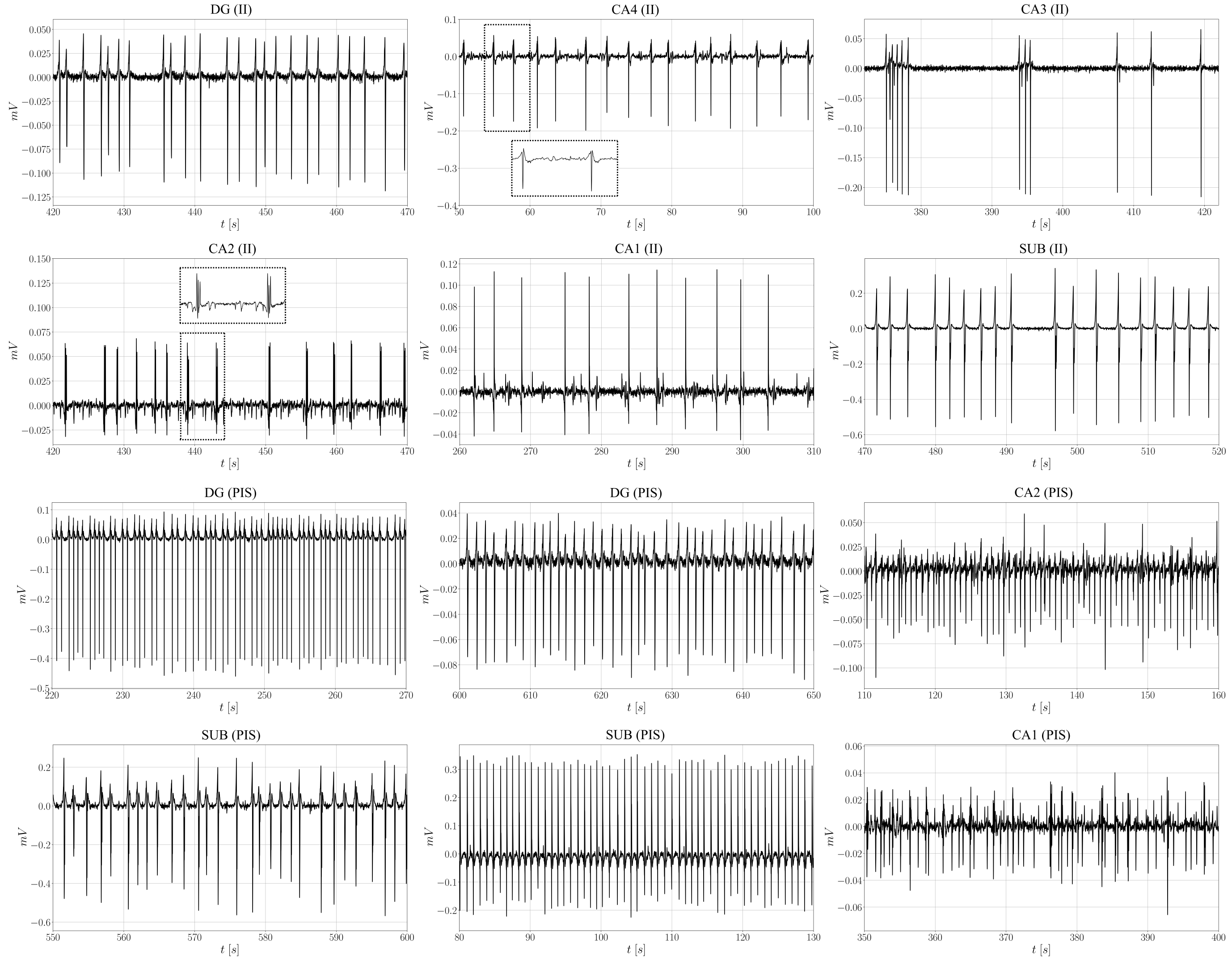

Fig A. Main structures found in the hippocampal subfields during the interictal (II) and ictal (PIS) states. Note that a more irregular and less frequent spiking pattern is observed for all selected epochs from II, whereas a more regular and frequent spiking pattern is observed for all selected epochs from PIS. All signals were properly filtered to reduce the effect of background or unrelated activity. A time window of 50 seconds is considered for all cases for purposes of visual comparison.

Adapting Neuron Dynamic Models to the Epileptiform Activity

As references for the behavior observed in the real signals analyzed in this work, we have chosen two commonly studied dynamic models: the Hindmarsh and Rose [1] and the Izhikevich [2] models. The main reasons for doing so are as follows: both intend to represent, to a certain extent, physiological activities that biological neurons undergo, such that their parameters may be potentially linked to physical factors; the first one was essential to understanding how bursting activity is generated by neurons [1], whereas the second one is a

rather flexible model able to represent a variety of spiking behaviors [2]; they are relatively simple and easily addressable, presenting three and two variables, respectively; at last, their simulations provide a fairly good representation for the structures covered in the previous section and are complementary, which also justifies the option for two models instead of only one. The Hindmarsh-Rose (HR) model is expressed by the following system [1]:

$$\begin{cases} \dot{x} = y - ax^3 + bx^2 + I - z \\ \dot{y} = c - dx^2 - y \\ \dot{z} = r[s(x - x_{rest}) - z] \end{cases} \tag{1}$$

where a, b, c and d are usually, but not necessarily, fixed parameters [1,3], which model the cubic and quadratic nullclines in the phase portrait of the system [1]. I is intended to represent the current applied to the neuron membrane, which either flows inward or outward the cell [1,3], r works as a time scale, controlling the speed of variation of the slow variable z, which in turn is related to the efficiency of slow channels in exchanging ions [3], and s is known as the adaptation variable [3]: lower values represent a weaker coupling with the membrane potential x, thus leading to a weaker accomodation and higher spiking [1,3], whereas higher values imply in stonger accomodation, leading to depolarizing after-potentials, for example [1]; y is known as a recovery variable, which, albeit not measurable, helps unfold the attractor related to the spiking behavior [1]; at last, x_{rest} is the resting potential of the system [1,3].

The Izhikevich (IZ) model, in turn, is given by [2]:

$$\begin{cases} \dot{v} = 0.04v^2 + 5v + 140 - u + I \\ \dot{u} = a(bu - v) \end{cases} \tag{2}$$

subjected to: if $v \geq 30$mV, then $v \leftarrow c$ and $u \leftarrow u + d$, where a, b, c and d are also usually fixed parameters, representing, respectively [2]: the time scale of the recovery variable u, the sensitivity of variable u to the subthreshold oscillations, the after-spike reset value of the membrane potential v, and the after-spike reset value of the recovery variable. Note that this model presents a jump in the time domain to account for the action potential; however, although relatively simpler than the first one, it is capable of reproducing many different neuron behaviors remarkably, as well as ensembles of neurons interacting with each other, when simulated as such [2].

To represent the dynamic structures present in the real signals I, b, r or s, from the Hindmarsh-Rose model, and I or d, from the Izhikevich model, can be varied while keeping the remaining ones constant. As demonstrated in recent works [3,4], each of these parameters may work as control variables, thus driving the

system into or out of a chaotic behavior. In practical terms, there is not only one possible biophysical factor responsible for providing more complexity, or chaoticity, to the system (in this case, neural tissues undergoing epileptiform activity).

In this work, for simplicity, we chose to analyze I, from the Hindmarsh-Rose model, and d, from the Izhikevich model, since their range of variation is conveniently large enough compared to the other ones, especially for the first model [3]. Nonetheless, other parameters have been tested and similar results can be obtained. Also, to better reproduce the amplitudes from Fig. 8, the responses were scaled and had their baselines shifted upwards.

Applying RQA to the Dynamic Models

Before applying RQA to the dynamic models, some considerations must be made. As simulated signals, their time scale is not equivalent to that verified in the real ones; that is, although their event ratio is similar, their unit of time (or time window) is not. Therefore, we considered, for simplicity, time windows that account for 1:3 and 2:3 ratios, as previously demonstrated. The RQA parameters were set to the ones used in the real signals: embedding dimension m = 5, line parameters of $l_{min} = 3$ and $v_{min} = 3$, and Theiler window of width 3 ($t_w = 3$). An exception is the time delay (set to 2), for the same reason as mentioned above. Nonetheless, as stated by [5], this is not a crucial parameter for RQA; also, the value chosen was able to unfold the attractor similarly to the real signals. Next, the following steps are adopted:

- different values for the control parameters of each model are tested in order to simulate the II and PIS states. For the Hindmarsh-Rose model, the range chosen was from I = 5.0 to I = 8.0, using a step of 0.3, which, although not clearly defined, falls in a transtion between activities [3]. For the Izhikevich model, the values of d are: -16, -15, -14, -13, -12.5, -12, -11.5, -11, -10, -9 and -8, which captures well the transition between chaotic to regular spiking, with an interface around -12 [4]. In this case, since each time window is analyzed separately, a multiple-stage step function (summing up to 11 cases) is used instead of the sigmoid one, such that one of these values is kept constant throughout the whole simulation to mimic the real signals;

- for each time window of the models, the RQA parameters investigated in this work are calculated. In total, to provide a good trade-off between number of samples and computational cost, 100 iterations are run for each of the 11 cases explained above. The algorithm applied belongs to the PyRQA library [6].

Analysis of RQA parameters for both models

Fig B presents complementary results for both computational models considering $\epsilon = 0.025$ and 0.05.

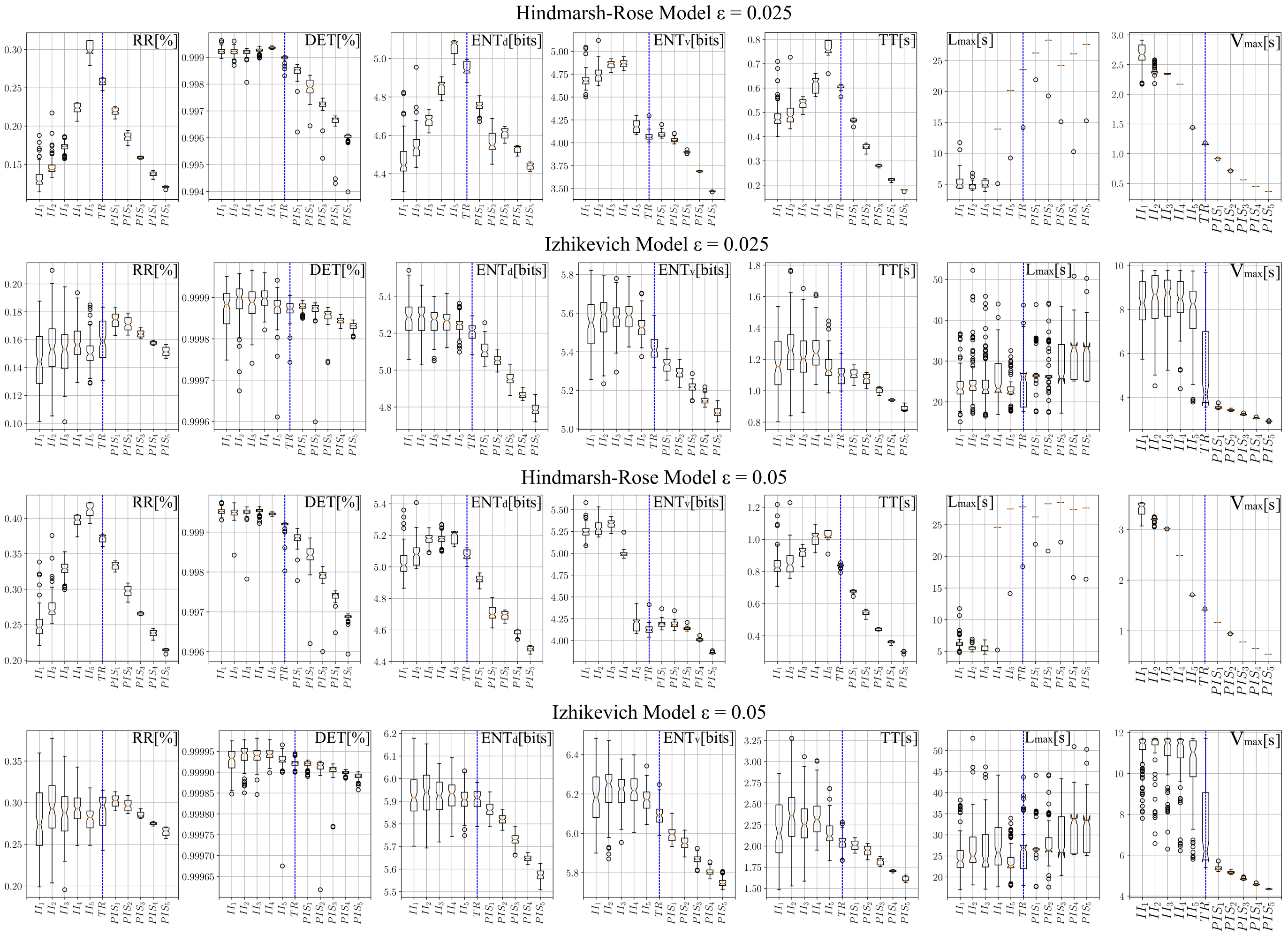

Fig B. Box plots for the six parameters studied in this work (an exception is the vertical entropy , which is a complementary result) for the 11 cases considering the Hindmarsh-Rosel and Izhikevich models and 2 different treshold values (ε = 0.025, 0.05): 5 for interictal activity (II), 1 transition (TR) and 5 for the ictal activity (PIS). Vertical blue dashed lines indicate a visual separation between the chaotic and regular spiking.

Biophysical meanings associated to the parameters of each model

Conceptually speaking, both models were designed to represent neuron firing and bursting, which are inherent brain activities, whose richness in behavior is desired in terms of information transmission at neuronal levels. Nevertheless, the HR model is at the core of recently proposed models that do mimic seizures, as the Epileptor [7,8], and the dynamics described by both, IZ and HR models, provide a good representation of nonlinear effects found in spiking patterns linked to general deterministic/chaotic systems.

The Hindmarsh-Rose, because it is modeled with 3 equations and thus more parameters, presents a more likely link to biophysical factors, whose change in behavior is briefly explained below:

Current I: as demonstrated in both models, the higher the input current applied to the neuron, the less chaotic/more regular its behavior becomes. However, it must stressed that, depending on the choice of I and the other control parameters together, this behavior may be the opposite [3,4].

Parameter r and variable z: based on the study of molluscan neurons [1], a general mechanism for burst generation is caused by the subthreshold inward current carried by Ca^{2+} ions, resulting from an interburst increasing depolarizing process; during the burst, the inflow of Ca^{2+} leads to a slow outward Ca^{2+} activated K^+ current. Therefore, z plays the role of this latter outward current, thus switching the model from quiescence to a firing behavior, i.e. z decreases at the benning of bursting and increases when it is stopped. Based on the bifurcation diagrams [3], the higher r is, the less chaotic the system becomes. This implies that, when r gets higher, variable z presents an even slower evolving behavior. Biophysically speaking: since z represents adaptation, if its behavior is relatively slower, this represents a reduced efficiency of the slow channels in exchanging ions.

Parameter s: its value couples variable z to the membrane potential x. According to its bifurcation diagrams [3], increasing or decreasing its values may generate a more chaotic behavior, depending on the choice of the other parameters. However, for moderate/standard currents I in this model, the general result is that higher s corresponds to more adaptation, leading to prolonged depolarizing afterpotentials and less frequent spikings, whereas lower values (lower adaptation) lead to more frequent neuron firings [1].

It must be stressed that, as neuron spiking models, both of them do not account for the physiology of ensembles of neurons. However, in sum, three main reasons are identified as possible epileptiform triggering

factors at a micro scale, which are simultaneously plausible due to the bifurcation diagrams [3]: increased input current into the neuron cells, decreased efficiency of the slow channels for ion exchange, or, assuming moderate/standard currents, lower adaptation values. Furthermore, all the analyses we were interested on the evaluation of the spiking dynamics and how nonlinear features emerge by modulating parameters of coupling.

Adherence of the Computational Models to the Electrophysiological Recordings

To assess the consistency of the results with respect to the similarity between real signals and artificial signals (produced by the two computational models), a preliminary analysis using autoregressive (AR) models was carried out. AR modeling is a relatively simple black-box technique that is traditionally applied to time series for purposes of system identification [9]. It involves a linear difference equation which aims to predict current values based on previous observations [9,10]:

$$y_k = \phi_0 - \sum_{i=1}^{K} \phi_i y_{k-i} + \varepsilon_k \tag{3}$$

where k indicates a discrete-time series, ϕ_i, $i = 1, ..., K$, are coefficients that weigh the past observations y_{k-i}, $i = 1, ..., K$ of variable y_k, ϕ_0 is the mean value of the time series, and ε_k is the error associated to the k discrete time. Considering a reference signal (in this case, an electrophysiological recording), the coefficients ϕ_i can be found through an optimization process involving a minimization function and the optimal least squares algorithm [9].

Such a modeling is often used in civil and mechanical engineering applications for purposes of structural health monitoring (SHM) [11-13]. Essentially, measurements of a vibrating structure are made in two main conditions: healthy and damaged (when a damage is present). Then, since the AR coefficients or the residuals of the model are sensitive to the presence of damage [12], classification can be performed. This is possible due to the fact that each coefficient represents a regression of the signal onto its delayed version, thus revealing how well past values determine future ones. In this work, the same concept is considered for the II and PIS signals. It is thus expected that ϕ_i can distinguish both activities because they contain information about the underlying brain dynamics.

For simplicity, only the first coefficient, ϕ_1, is adopted (while keeping $\phi_0 = 0$ as the baseline), but as a random variable; that is, we reconstructed several 10-second epochs (same duration applied for the RQA analysis) of each signal and saved their first coefficients. This procedure was done for all 22 real II and 13 real PIS recordings, as well as the artificial signals, considering the HR and IZ models. The total duration of the simulated models was determined based on the longest real signal, but the epochs were also equal to 10 seconds. Moreover, different levels of noise, from 0.05% to 10% (in 20 steps of 0.05% each), were added to the artificial signals for further comparisons. The vector of noise is represented by a zero-mean unity-variance normal distribution multiplied by such levels.

Fig C presents the AR coefficients, in terms of confidence intervals, related to the probabilities of class prediction (II or PIS) using logistic regression [14], where the AR coefficients of real signals (II and PIS) were used as projectors for the classification of artificial signal coefficients, following:

$$\ln\left(\frac{\pi_j}{\pi_r}\right) = \beta_{j0} + \beta_{j1}X_{j1} + \beta_{j2}X_{j2} + ... + \beta_{jp}X_{jp}, \quad j = 1, ..., k-1 \tag{4}$$

where π stands for a categorical probability, r corresponds to the reference category, β are the coefficients, X are the predictions and k is the last category (in this case $k = 2$). Here, we used the "mnrfit" Matlab® function for nominal models, considering all coefficients of all HP subfields for II and PIS to construct the projective basis. Afterward, we projected separately the artificial signal's coefficients, labeled as II and PIS activities, to quantify the probability of being classified as II or PIS. The mean and confident intervals were considered for different trials (> 50) and for each noise level (0,05% to 10%).

The results show that the coefficients effectively recognized and distinguished between the two main patterns, II and PIS, mainly the HR model. By considering low levels of noise, we observed that the IZ model produces misclassifications, but as it increases the performance gets better. We must consider that these effects might happen, firstly because the models do not reflect the whole complexity of real signals, and secondly because by using only the first AR coefficient we may neglect important information required for a better classification performance using logistic regression. In addition, considering that they reflect sufficiently well the underlying brain dynamics, the ranges of noise may indicate different levels of background activity in which the dynamics of each signal is present.

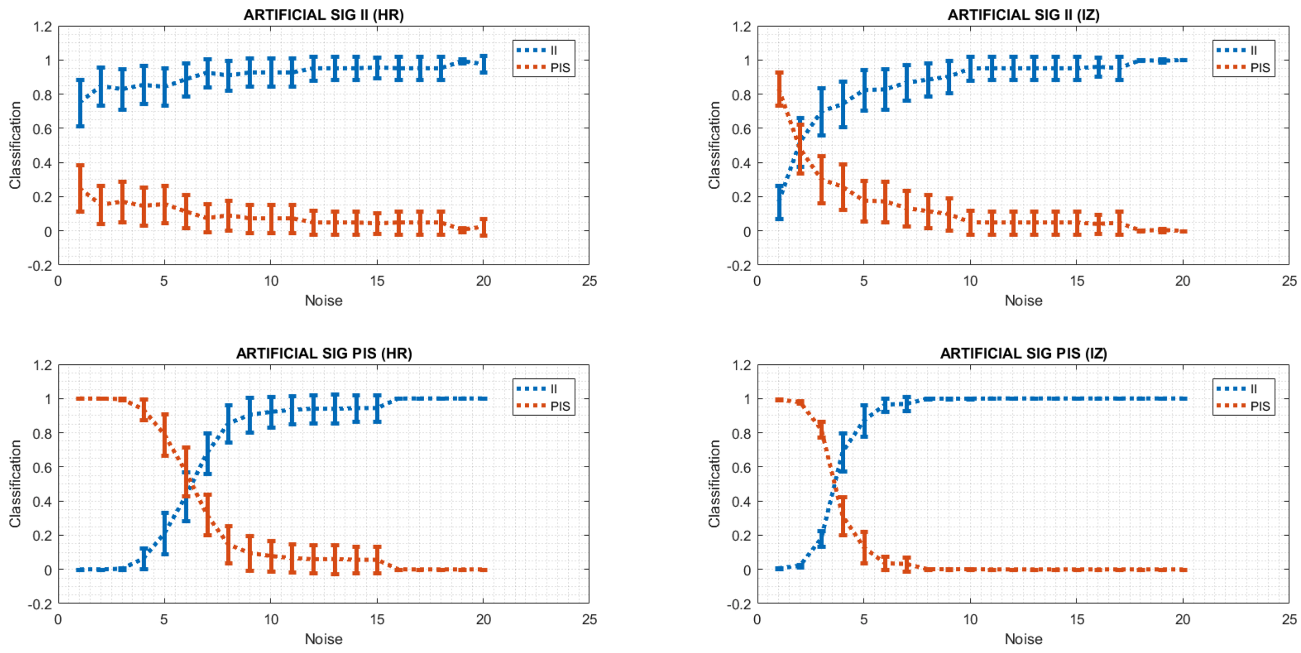

Fig C. Logistic regression to classify the computational models in II and PIS conditions based on the AR coefficients obtained from real signals. The first picture (left above: II signals of HR model) indicates in blue that the artificial coefficients labeled as II activities were correctly classified as II patterns, whereas orange color indicates that the artificial coefficients from II activities were incorrectly classified as PIS patterns. The second picture (left below: PIS signals for HR model) indicates in orange that the artificial coefficients labeled as PIS activities were correctly classified as PIS patterns, whereas blue color indicates that the artificial coefficients from PIS activities were incorrectly classified as II patterns. The pictures showed right above and right below represent the same analysis for the IZ model. The curves are plotted considering their probability of correct classification related to their confidence intervals at 95% confidence, for > 50 samples. Noise varies from 0% to 10% in 20 steps of 0.05% each.

Fig D provides a counterproof, where multiple comparisons between real signals (II and PIS) and their reconstructed models were made and their root mean square errors (RMSE) were calculated, presented in terms of confidence intervals. For this situation, it is expected that comparisons of the type: real PIS signal vs reconstructed PIS signal based on PIS coefficients provide a lower error than their reconstructions using II coefficients, for example, and vice-versa. Note that this indeed holds true for PIS signals, but not for II signals.

The miscorrespondence presented mainly in II signals and AR models may be justified since the “a priori” categorization of PIS and II was made using only the criterion of event rates (interictal-like events (II) present $r < 40/min$, and periodic ictal spiking (PIS) presents $r > 40/min$). However, as we have shown there are other statistical and physical properties under these two periods (or conditions) underlying these activities. Therefore, some misclassification most probably has happened using only this linear criterion, and it was

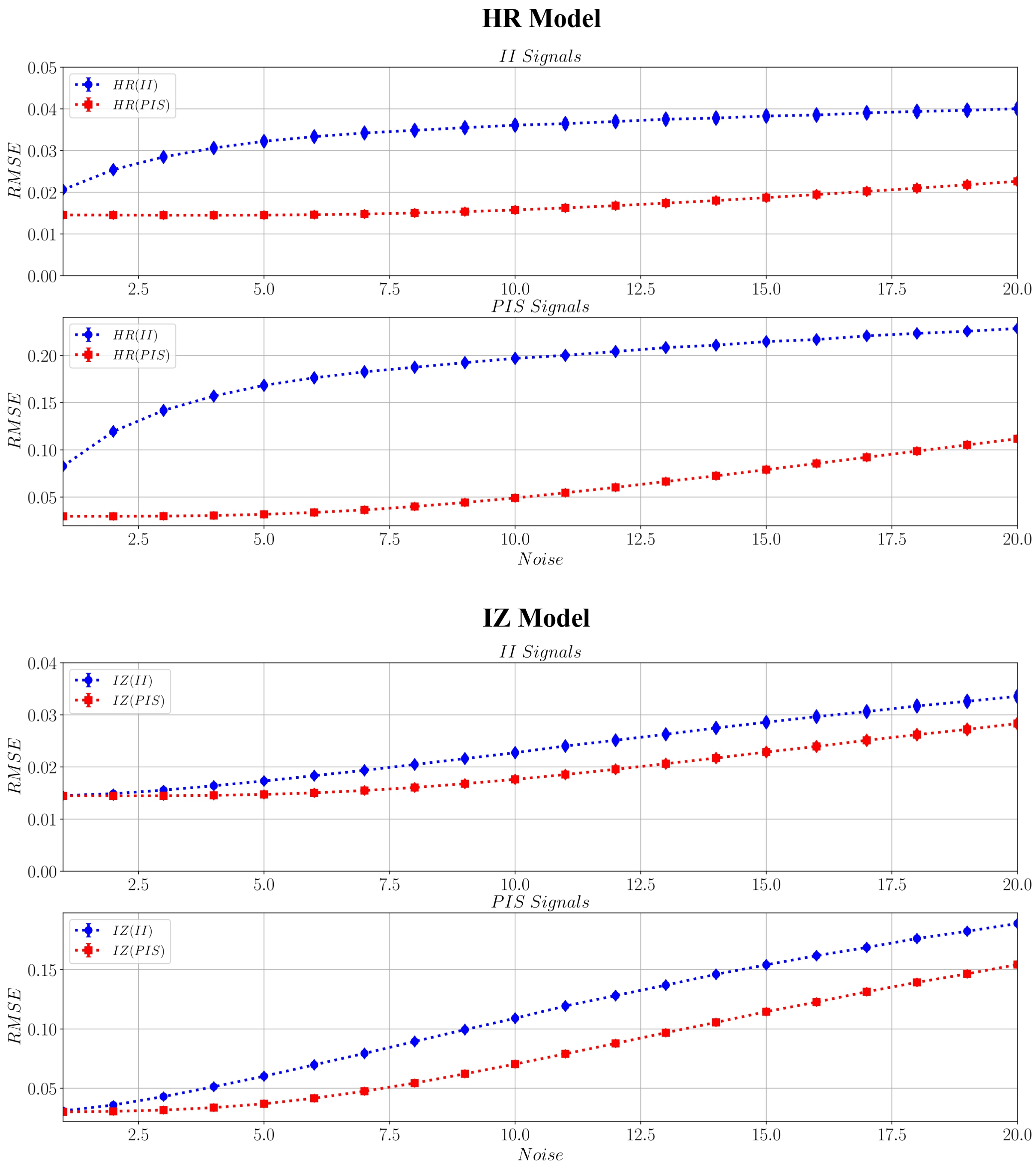

Fig D. Root Mean Square Error (RMSE) between combinations of real II/PIS activities and their reconstructions using II/PIS coefficients (confidence intervals). The titles indicate which group of real signals are compared (II or PIS). The legends indicate which types of coefficients were used to reconstruct them and the respective computational models. Blue dots indicate reconstructions using II coefficients; red squares indicate reconstructions using PIS coefficients. The curves are plotted considering their confidence intervals at 95% confidence. Noise varies from 0% to 10% in 20 steps of 0.05% each.

accused by the introduction of noise in the computational models. Nonetheless, it is important to emphasize that these computational models are not able to capture all the statistical features related to the real signals.

Besides, II patterns, as the supposed corresponding models in chaotic conditions, present a much richer spectrum of spiking behavior. That is, epochs from II/simulated chaotic activities have different irregular patterns, thus leading to a higher variance of AR coefficients used for regression. In this way, reconstructions of PIS activities are favored. This effect can be better illustrated in Fig E, where the results from Fig. D are shown, but now as boxplots plotted in log scale. Note that the same tendency is present for both models: while PIS signals reconstructed with PIS coefficients have a lower variance and error, their reconstructions with II coefficients have higher number of outliners and variance; on the other hand, II signals reconstructed with PIS and II coefficients have fairly similar medians, but the variance for II is slightly higher.

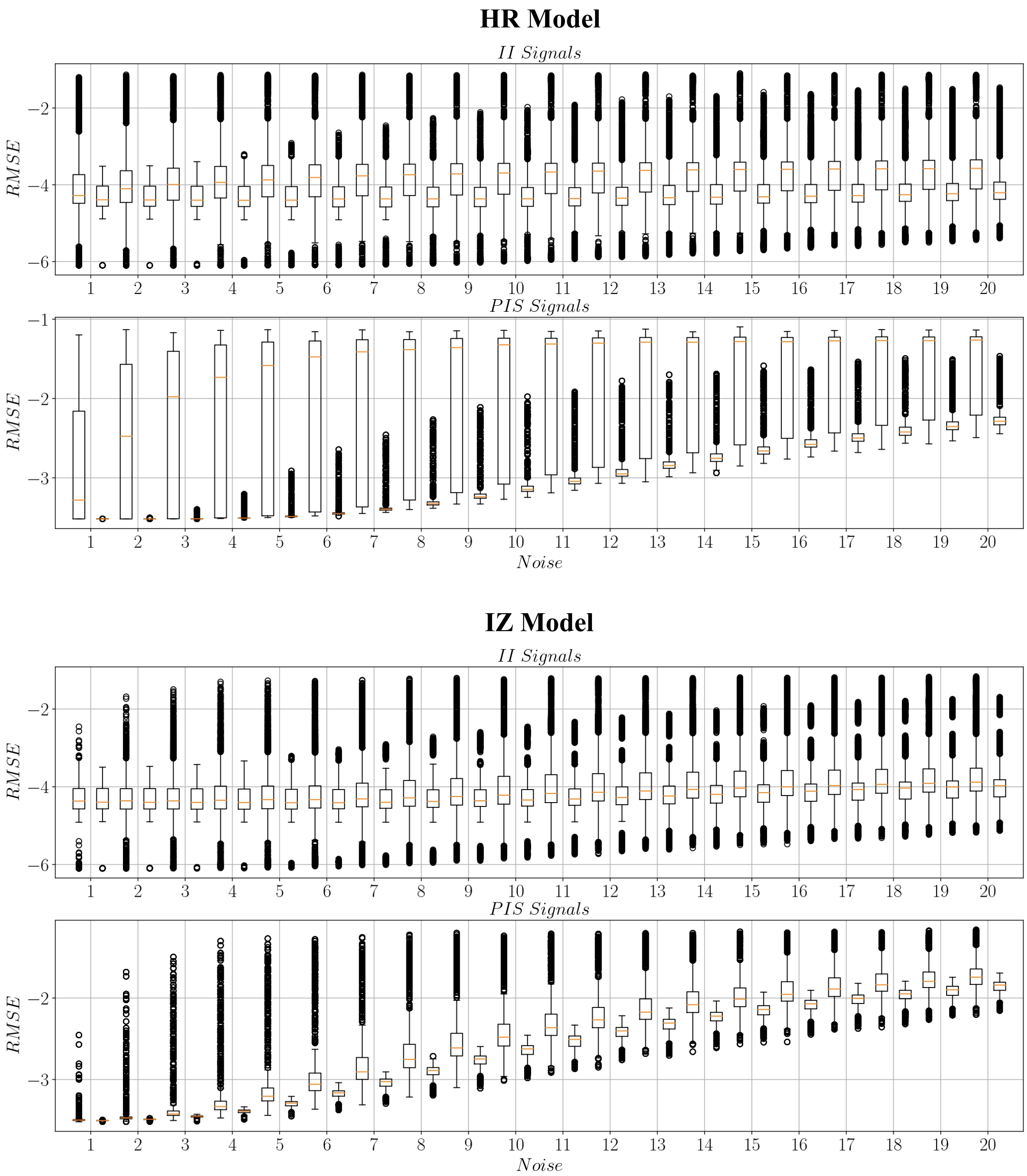

Fig E. Root Mean Square Error (RMSE) between combinations of real II/PIS activities and their reconstructions using II/PIS coefficients (boxplots). The titles indicate which group of real signals are compared (II or PIS). The curves are plotted considering boxplots in a log scale: to the left, boxplots of reconstructions using II coefficients; to the right, the same but for PIS coefficients. Noise varies from 0% to 10% in 20 steps of 0.05% each.

One limitation of this analysis is the fact that AR modeling is a linear technique and usually requires time series to be stationary [15]. However, fairly good comparisons could be carried out in this work, suggesting that

computational models can theoretically and minimally reflect real patterns. Further work is intended to obtain a more comprehensive analysis of the adherence of AR models to real signals, especially considering more coefficients or other alternatives, such as the ARMA (autoregressive moving average), ARIMA (autoregressive integrated moving average) or NARMA (nonlinear autoregressive moving average) models [16-20].

References

1. Hindmarsh JL, Rose RM. A model of neuronal bursting using three coupled first order differential equations. P Roy Soc Lond B Bio. 1984;221(1222): 87-102.

2. Izhikevich EM. Simple model of spiking neurons. IEEE T Neural Networ. 2003;14(6): 1569-72.

3. Storace M, Linaro D, de Lange E. The Hindmarsh–Rose neuron model: bifurcation analysis and piecewise-linear approximations. Chaos. 2008;18(3): 033128.

4. Nobukawa S, Nishimura H, Yamanishi T, Liu JQ. Analysis of chaotic resonance in Izhikevich neuron model. PloS One. 2015;10(9): e0138919.

5. Webber Jr CL, Zbilut JP. Recurrence quantification analysis of nonlinear dynamical systems. Tutorials in contemporary nonlinear methods for the behavioral sciences. 2005;94(2005):26-94.

6. Rawald T, Sips M, Marwan N. PyRQA—Conducting recurrence quantification analysis on very long time series efficiently. Comput Geosci. 2017;104:101-108.

7. Jirsa VK, Stacey WC, Quilichini PP, Ivanov AI, Bernard C. On the nature of seizure dynamics. Brain. 2014;137(8): 2210-2230.

8. Proix T, Bartolomei F, Chauvel P, Bernard C, Jirsa VK. Permittivity coupling across brain regions determines seizure recruitment in partial epilepsy. J Neurosci. 2014;34(45): 15009-15021.

9. Ljung L. System identification: theory and practice for the user. Upper Saddle River (NJ): Prentice Hall; 1987.

10. Durbin J, Koopman SJ. Time series analysis by state space methods. Oxford: Oxford university press; 2012.

11. Yao R, Pakzad SN. Autoregressive statistical pattern recognition algorithms for damage detection in civil

structures. Mech Syst Signal Process. 2021;31: 355-368.

12. Entezami A, Shariatmadar H. An unsupervised learning approach by novel damage indices in structural health monitoring for damage localization and quantification. Struct Health Monit. 2018;17(2): 325-345.

13. Yang JH, Lam HF. An innovative Bayesian system identification method using autoregressive model. Mech Syst Signal Process. 2019;133: 106289.

14. MathWorks® [Internet]. United States: The MathWorks, Inc.; 1994-2022. Multinomial Logistic Regression; 2022 [Cited 2022 Mar 26]; [about 10 screens]. Available from: https://www.mathworks.com/help/stats/mnrfit.html.

15. Fox J. Applied regression analysis and generalized linear models. Sage Publications; 2015.

16. Landau ID, Karimi A. A recursive algorithm for ARMAX model identification in closed loop. IEEE T Automat Contr. 1999;44(4): 840-843.

17. Jansson M. Subspace identification and ARX modeling. IFAC Proceedings Volumes. 2003;36(16): 1585-1590.

18. Baldacchino T, Anderson SR, Kadirkamanathan V. Computational system identification for Bayesian NARMAX modelling. Automatica. 2013;49(9): 2641-2651.

19. Liu H, Zhu L, Pan Z, Bai F, Liu Y, Liu Y, et al. ARMAX-based transfer function model identification using wide-area measurement for adaptive and coordinated damping control. IEEE T Smart Grid. 2015;8(3): 1105-1115.

20. Retes PFL, Aguirre LA. NARMAX model identification using a randomised approach. Int J Model Identif. 2019;31(3): 205-216.
